# Ion Channel Expression and Electrophysiology of Singular Human (Primary and Induced Pluripotent Stem Cell-Derived) Cardiomyocytes

**DOI:** 10.3390/cells10123370

**Published:** 2021-11-30

**Authors:** Christina Schmid, Najah Abi-Gerges, Michael Georg Leitner, Dietmar Zellner, Georg Rast

**Affiliations:** 1 Drug Discovery Sciences, Boehringer Ingelheim Pharma GmbH & Co. KG, 88397 Biberach, Germany; michael_2.leitner@boehringer-ingelheim.com (M.G.L.); georg.rast@boehringer-ingelheim.com (G.R.); 2 Food Chemistry and Toxicology, Department of Chemistry, University of Kaiserslautern, 67663 Kaiserslautern, Germany; 3 AnaBios Corporation, San Diego, CA 92109, USA; najah.abigerges@anabios.com; 4 Non-Clinical Statistics, Boehringer Ingelheim Pharma GmbH & Co. KG, 88397 Biberach, Germany

**Keywords:** cardiomyocytes, primary human cardiomyocytes, human induced pluripotent stem cell-derived cardiomyocytes, ion channel, gene expression, electrophysiology

## Abstract

Subtype-specific human induced pluripotent stem cell-derived cardiomyocytes (hiPSC-CMs) are promising tools, e.g., to assess the potential of drugs to cause chronotropic effects (nodal hiPSC-CMs), atrial fibrillation (atrial hiPSC-CMs), or ventricular arrhythmias (ventricular hiPSC-CMs). We used single-cell patch-clamp reverse transcriptase-quantitative polymerase chain reaction to clarify the composition of the iCell cardiomyocyte population (Fujifilm Cellular Dynamics, Madison, WI, USA) and to compare it with atrial and ventricular Pluricytes (Ncardia, Charleroi, Belgium) and primary human atrial and ventricular cardiomyocytes. The comparison of beating and non-beating iCell cardiomyocytes did not support the presence of true nodal, atrial, and ventricular cells in this hiPSC-CM population. The comparison of atrial and ventricular Pluricytes with primary human cardiomyocytes showed trends, indicating the potential to derive more subtype-specific hiPSC-CM models using appropriate differentiation protocols. Nevertheless, the single-cell phenotypes of the majority of the hiPSC-CMs showed a combination of attributes which may be interpreted as a mixture of traits of adult cardiomyocyte subtypes: (i) nodal: spontaneous action potentials and high HCN4 expression and (ii) non-nodal: prominent I_Na_-driven fast inward current and high expression of SCN5A. This may hamper the interpretation of the drug effects on parameters depending on a combination of ionic currents, such as beat rate. However, the proven expression of specific ion channels supports the evaluation of the drug effects on ionic currents in a more realistic cardiomyocyte environment than in recombinant non-cardiomyocyte systems.

## 1. Introduction

Human induced pluripotent stem cell-derived cardiomyocytes (hiPSC-CMs) constitute a promising tool for basic science, regenerative medicine, and drug development [1]. Some applications necessitate the availability of ideally homogenous hiPSC-CM cell culture models representing specific cellular subtypes that in the adult human heart can be classified into nodal, atrial, and ventricular cardiomyocytes based on anatomical and functional differences. During safety assessment in drug development, for example, nodal hiPSC-CMs may be used as a model for determining the chronotropic effects of compounds; atrial hiPSC-CMs may facilitate research on atrial fibrillation; and ventricular hiPSC-CMs may provide a suitable model for pro-arrhythmic risk evaluation (i.e., estimating the risk for the ventricular “Torsade de Pointes” (TdP) type arrhythmia).

Importantly, the primary cardiomyocyte subtypes largely (but not exclusively) differ in expression and characteristics of ion channels that in turn critically determine the cells’ significantly different physiology and functions [2]. The pump function of the heart is maintained by the atrial and ventricular cells, whereas the smaller nodal cardiomyocytes of the sinoatrial node generate spontaneous action potentials that naturally control excitation of the heart [2]. It is widely accepted that in nodal cells membrane-derived processes, but also cellular Ca^2+^ dynamics—referred to as a membrane clock or a Ca^2+^ clock, respectively—contribute to the periodic membrane potential depolarizations [3]. The activity of three ion channel isoforms mainly contributes to the membrane clock mechanism: HCN4 (mediating the funny current I_f_); CACNA1G (encoding T-type calcium channel Ca_V_3.1 mediating I_CaT_); and CACNA1D (encoding L-type calcium channel Ca_V_1.3 mediating I_CaL_) [4,5,6,7]. It is noteworthy that these three channel isoforms are also expressed in the atrial cardiomyocytes, but no (or only rather low) expression of the genes encoding these channels was detected in the primary ventricular cardiomyocytes [2]. The spontaneous activity of primary nodal cardiomyocytes is further facilitated by the absence of the otherwise silencing inward rectifier potassium current I_K1_ (KCNJ2 and KCNJ4, encoding Kir2.1 and Kir2.3, respectively) that is abundant in the other excitable cardiac cell types [2,8]. Transcripts of the SCN5A gene (encoding Na_V_1.5 channel subunits) are present in both the primary atrial and the ventricular cardiomyocytes and are responsible for the fast inward sodium current I_Na_ and the action potential upstroke, while no abundance of SCN5A was reported in the primary cells of the central sinoatrial node [9] Whereas the expression of KCNA5 (encoding K_V_1.5 which conducts the ultra-rapid delayed rectifier potassium current I_Kur_) is highly associated with primary atrial cardiomyocytes and the expression of CACNA1C (encoding L-type calcium channel Ca_V_1.2 which conducts I_CaL_), and KCNH2 (encoding the K_V_11.1/hERG channel underlying the rapid delayed rectifier potassium current I_Kr_) is reported in all three cardiomyocyte subtypes of the heart [2].

Subtype-specific hiPSC-CM models may be generated by enrichment or separation of a specific subtype from non-directed cardiac differentiation approaches or by subtype-directed differentiation [10]. Indeed, several hiPSC-CM models are commercially available, including iCell cardiomyocytes (Fujifilm Cellular Dynamics, Madison, WI, USA) and atrial Pluricytes or ventricular Pluricytes (Ncardia, Gosselies, Charleroi, Belgium). Atrial and ventricular Pluricytes may represent chamber-specific models [11], but clear evidence for this discrimination is currently not available as the data supporting this hypothesis—especially for the atrial Pluricytes—are missing. The iCell cardiomyocytes (non-directed approach) have been assessed by the Comprehensive in vitro Proarrhythmia Assay (CiPA) initiative [12,13] and are apparently a mixture of spontaneously and electrically active nodal-, atrial-, and ventricular-like cardiomyocytes [14]. In fact, we observed that iCell cultures contain spontaneously beating, but also quiescent, cardiomyocytes, suggesting that these cultures may comprise at least two cardiac subtypes, i.e., spontaneously beating cells resembling nodal cardiomyocytes and intrinsically quiescent cells potentially representing ventricular or atrial cardiomyocytes. However, the cellular identity and the molecular determinants of the functionally different iCell cardiomyocytes are incompletely understood, and it remains unknown whether these subtypes may be separated from each other to create subtype-specific cardiomyocyte models.

Despite this clear evidence for the heterogeneity of hiPSC-CM cultures, only limited single-cell data are available. Previous studies addressed the expression of certain cardiac channel isoforms at the population level together with the demonstration of certain cardiac ionic currents in single cells (e.g., for SCN5A/I_Na_, CACNA1C/I_CaL_, and KCNH2/I_Kr_) [15,16,17,18]. However, they utilized different subsets of cells for the investigation of bulk ion channel expression and single-cell electrophysiology (ionic current and action potential measurements), making it impossible to make conclusions about the actual cellular identities, i.e., the combination of ion channels and ionic currents in individual cells. Our study therefore aimed to clarify the characteristics and chamber specificity of commercially available hiPSC-CMs (iCell cardiomyocytes, atrial and ventricular Pluricytes) at the single-cell level by assessing multiple parameters in individual cells. Utilizing a single-cell reverse transcriptase quantitative polymerase chain reaction (RT-qPCR) technique in combination with whole cell patch clamp, we recorded electrophysiological parameters (spontaneous beat rate, capacitance, and sodium-driven fast inward current) and assessed the expression levels of nine cardiac ion channel transcripts associated withknown subtypes of adult human cardiomyocytes (HCN4, CACNA1G, CACNA1D, KCNA5, KCNJ4, SCN5A, KCNJ2, CACNA1C, and KCNH2). We compared spontaneously beating and non-beating iCell cardiomyocytes with respect to electrophysiology and ion channel expression to unravel the composition of the iCell cardiomyocyte culture. We contrasted the electrophysiology data and ion channel expression between iCell cardiomyocytes and atrial and ventricular Pluricytes to assess the chamber specificity of the hiPSC-CM models in comparison to primary atrial and ventricular cells isolated from human donor hearts. In addition, electrophysiological and transcriptomic data of the same cell were used to investigate the correlation between the ion channel transcripts and the electrophysiological parameters. We found that beating and non-beating iCell cardiomyocytes did not represent the nodal, atrial, or ventricular cells of the adult human heart. While our findings point to a trend towards chamber specificity of the atrial and ventricular Pluricytes with respect to ion channel mRNA expression, the majority of all three commercially available cell models are dissimilar to mature human primary cardiomyocytes. They show unexpected combinations of ion channel transcripts and electrophysiological parameters on the single-cell level, combining characteristics of nodal, atrial, and ventricular cardiomyocytes. The apparent lack of subtype specificity clearly limits the applicability of certain hiPSC-CM cultures in research and drug development but at the same time allows for the analysis of several cardiac ion channels in a close to native environment.

## 2. Materials and Methods

### 2.1. Culture of hiPSC-Derived Cardiomyocytes

The iCell cardiomyocytes (product number 01434, Fujifilm Cellular Dynamics, Madison, WI, USA) were cultured as described previously [19]. The ventricular (product number PCK-1.5, Ncardia, Gosselies, Charleroi, Belgium) and atrial Pluricytes (custom production, Ncardia, Gosselies, Charleroi, Belgium) were thawed and cultured according to the manufacturer’s instructions (for details see [19]). The aim of our study was to provide a snapshot of single-cell profiling at a relevant age for users of the cells to enable conclusions for experiments, rather than investigating the maturation of cells in a longitudinal study. The cells were singularized before the experiments for 12–39 days (iCell cardiomyocytes), for 7–20 days (atrial Pluricytes), or for 13–23 days (ventricular Pluricytes) after thawing. The iCell cardiomyocytes originated from 5 different vials of the same lot, the ventricular Pluricytes from 3 vials of the same lot, and the atrial Pluricytes from 2 vials of the same lot.

### 2.2. Singularizing hiPSC-Derived Cardiomyocytes

To investigate single cells, the singularization of monolayer hiPSC-CM cultures was performed by splitting the confluent cells of a single well into 4–6 new wells of a 24-well plate, as described previously [19,20]. Between day 1 and 3 after singularizing, the hiPSC-CMs were used for experiments.

### 2.3. Procurement of Donor Hearts

The procurement of donor hearts and the isolation of primary human cardiomyocytes were performed at the AnaBios Corporation (San Diego, CA, USA). All methods were carried out in accordance with the relevant guidelines and regulations. All human hearts used for this study were non-transplantable and ethically obtained by legal consent (first person or next-of-kin) from cadaveric organ donors in the United States. AnaBios’ recovery protocols and in vitro experimentation were pre-approved by IRBs (Institutional Review Boards) at transplant centers within the US OPTN (Organ Procurement Transplant Network). Furthermore, all transfers of the donor hearts are fully traceable and periodically reviewed by US Federal authorities. Donor characteristics, heart number, and donor identifier are shown in Supplementary Table S1, and the exclusion criteria were previously described [21].

### 2.4. Isolation and Storage of Primary Human Cardiomyocytes

Upon arrival at the laboratory, the hearts were re-perfused with ice-cold proprietary cardioplegic solution, and the adult human primary ventricular and atrial myocytes were isolated enzymatically from the ventricles or atria, respectively [21,22]. Single-cell suspensions were stored in a proprietary storage solution at 4 °C until use. The primary ventricular cardiomyocytes were used 1–5 days after isolation, and the atrial cardiomyocytes were used 1–4 days after isolation by trickling cell solution on laminin-coated (24 h at 4 °C or 2 h at room temperature) glass coverslips and letting them attach for 10 min at room temperature.

### 2.5. Electrophysiological Recordings and Cell Harvesting

All the procedures were performed ideally under ribonuclease (RNase)-free conditions. For the technical details and solutions, see [20]. The randomized selection of single cardiomyocytes was accomplished by picking the central viable cell of an arbitrarily chosen field of view under an inverted CKX41 microscope (Olympus, Tokyo, Japan). Before starting the electrophysiological measurements, the spontaneous beating frequency of the hiPSC-derived cardiomyocytes was counted visually for 30 s. Electrophysiological recordings were performed on the hiPSC-CMs immediately after establishment of the whole-cell configuration of the patch-clamp technique. All electrophysiological measurements were performed at 32 °C with an EPC10-USB patch clamp amplifier controlled with PatchMaster software (HEKA, Lambrecht, Germany). The experiments were performed as fast as possible to avoid both the extensive diffusion of cellular mRNA into the patch pipette and mRNA degradation. This procedure rendered detailed analysis of currents (i.e., voltage dependence, kinetics, etc.) or pharmacological investigations (i.e., measurements of pharmacologically activated currents such as the acetylcholine-induced K+ current I_KACh_) impossible. The cells were kept in extracellular buffer throughout the recordings (137.0 mM NaCl; 2.7 mM KCl; 8.0 Na_2_HPO_4_; 2.0 mM KH_2_PO_4_; 0.5 mM MgCl_2_∙6 H_2_O; 0.9 mM CaCl_2_; and 5.0 mM glucose). Patch pipettes were pulled from borosilicate glass (Hilgenberg, Malsfeld, Germany) and had a membrane resistance of 2.3–3.5 MΩ after filling with intracellular solution (for experiments with hiPSC-CMs: 130.0 mM K-aspartate; 5.0 mM MgCl_2_∙6 H_2_O; 5.0 mM EGTA; 4.0 mM K_2_ATP; 10.0 mM HEPES; and pH 7.2 with KOH. For experiments with primary cardiomyocytes: 100.0 mM K-aspartate; 25.0 mM KCl; 1.0 mM MgCl_2_∙6 H_2_O; 10.0 mM EGTA; 5.0 mM K_2_ATP; 5.0 mM HEPES; and pH 7.2 with KOH). To record the fast inward current, the cells were clamped at a holding potential of −100 mV, followed by a depolarizing voltage step to −30 mV (for 100 ms every 3 s). The current and voltage signals were Bessel filtered at 10 kHz before being digitized at 20 kHz. The R_s_ was compensated throughout the recording (typically 50%). The cell capacitance was determined via the intrinsic compensation circuitry of the patch clamp amplifier. The inward current peak amplitudes were obtained by subtracting holding current at −100 mV from the inward peak current at −30 mV. In separate experiments (see Supplementary Figure S1a), we applied the specific inhibitor of voltage-dependent Na^+^ (Na_V_) channels, tetrodotoxin (TTX, 30 µM; Alomone, Jerusalem, Israel), and the Cal^2+^ (Ca_L_) channel blocker nifedipine (10 µM, Sigma-Aldrich, St. Louis, MO, USA) to the iCell cardiomyocytes. Action potential recordings were obtained for the ventricular Pluricytes by a “gentle switch” to current-clamp mode at a holding potential of −80 mV, i.e., the command current in current clamp mode was automatically set to the average value determined in voltage clamp mode for the holding potential. To ensure comparability of action potentials, the command current was adjusted to keep the cells in a range of −76 mV to −84 when necessary. The action potentials of ventricular Pluricytes were triggered by injecting rectangular current pulses (500–1000 pA for 2–3 ms) every 3 s.

Three replicate current and voltage traces per cell were exported to Microsoft Excel (Microsoft, Redmond, WA, USA) for analysis. The following electrophysiological parameters were determined: Capacitance [pF], beat rate [bpm], fast inward current amplitude [nA] and fast inward current density (current normalized to cell capacitance; [pA/pF]), upstroke velocity of action potential (AP) [V/s], and APD90 (AP duration at 90% repolarization, for calculation see Equation (1)) [ms].

APD90 = t _[at V 90% repolarisation]_ − t _[at max. AP upstroke velocity]_ (1)

with V _[90% repolarization]_ = V _[action potential amplitude]_ × 0.1 + V _[mean baseline before start of AP]._

Graphical representations were generated using GraphPad Prism (GraphPad software, San Diego, CA, USA). After electrophysiological measurements, the micropipette (filled with IC-solution) used for the recordings was gently removed from the cell, allowing the cell to re-seal. A large-bore pipette filled with of RNase-free phosphate buffered saline (PBS, approximately 1 µL) (Invitrogen, Carlsbad, CA, USA) was used to aspirate the same cell under visual control. After aspiration, the tip of the pipette was broken into a single nuclease-free 0.2 mL PCR-tube (Eppendorf AG, Hamburg, Germany) to subsequently perform a single-cell RT-qPCR. One coverslip with cardiomyocytes was used for a maximum of 30 min. In total, 72 iCells, 24 atrial Pluricytes, 29 ventricular Pluricytes, 40 primary atrial human cardiomyocytes, and 90 primary ventricular human cardiomyocytes were collected. It was not possible to determine every parameter for every single cell for technical reasons; therefore, *n* may vary between parameters.

### 2.6. Single-Cell RT-qPCR

Single-cell mRNA expression was determined in hiPSC-CMs and adult human primary cardiomyocytes, utilizing Ambion’s Single Cell-to-CT kit (Thermo Fisher Scientific, Waltham, MA, USA) in combination with sequence-specific TaqMan gene expression assays (Thermo Fisher Scientific, Waltham, MA, USA, Supplementary Table S7). The experiments were performed according to the manufacturer´s instructions (for details see [20]). The qPCR of every target gene was performed in single reactions as technical duplicates using the Roche Lightcycler 480 (Hoffmann-La Roche, Basel, Switzerland). A no-template-control (w/o cDNA) was included in all experiments as the negative control. The CT values (inversely proportional to the number of mRNA molecules on the log2 scale) were obtained using the 2nd-derivation method.

For technical reasons (including sample volume and plate format), the single-cell RT qPCR analysis was limited in its number of target genes. The analyzed genes of interest included two reference genes to confirm the successful aspiration of the cell under investigation (the structural cardiac gene TNNT2; GAPDH) and nine cardiac ion channels (HCN4, CACNA1G, CACNA1D, KCNA5, KCNJ4, SCN5A, KCNJ2, CACNA1C, and KCNH2). The nine ion channel transcripts were carefully selected considering the established expression in specific cardiac cell types of the mature human heart (nodal, atrial, and ventricular [2]) and their physiological and clinical relevance. However, selection of target genes implies that the conclusions we make are based on the investigated genes and not on the whole phenotype. Samples not showing expression of TNNT2 or GAPDH were excluded from further analysis. In the case that a CT value was given the tag “detector call uncertain” (often the case for very high CT values) or in the case of atypical amplification curves, this CT was replaced by the value of the technical replicate (if available and valid) or excluded from the analysis (if the replicate was invalid as well). In the case that the Lightcycler 480 did not detect a signal, the reactions were indicated with “CT = 0”. The highest CT value reported by the instrument for our experiments was “CT = 35”, tagged with the note “Late Cp call (last five cycles) has higher uncertainty”. It was reported previously that very high CT values may not be reliable and that the CT value corresponding to a single target cDNA molecule is usually 35–37 cycles for micro titer plates [23]. Therefore, we set all “CT = 0” values to “CT = 35” and defined a value of CT = 35 as a “negative” result (no detectable expression). Accordingly, the percentage of “positive cells” was calculated for binary analysis. For further quantitative, statistical analysis, CT values of CT = 35 were excluded. A more accurate method would have been to determine a standard curve for every target gene and derive the slope (efficiency) and y intercept (detection limit). However, determining standard curves for single-cell experiments is challenging, as the calibrator sample should consist of the same complex biological matrix and be processed in exactly the same way as the experimental samples. As we did not determine the standard curves or efficiencies, we performed downstream analysis using CT values and did not calculate the numbers of transcripts. Moreover, we did not compare between different ion channels as we cannot assure that the amplification efficiencies were comparable between different targets. Consequently, we calculated the mean CT and standard deviation (SD) for the positive cells as a surrogate for the expression level. Not surprisingly for cells of different origins, we recognized that primary cardiomyocytes have overall lower CT values (higher expression levels) for every target gene investigated, except HCN4 and CACNA1G, than the three hiPSC-CM groups (see Table 3). The reason may be that primary cardiomyocytes are larger than hiPSC-CMs. However, individual normalization of single-cell data is generally not recommended by the manufacturer [24] or the literature [23]. Nevertheless, in order to compare expression between the five cell groups, despite their different expression levels, we normalized each individual CT value for a specific ion channel with respect to the mean CT value of the GAPDH and TNNT2 of the whole-cell group. We calculated ΔCT values for every cell and target gene by subtracting the averaged CT of TNNT2 and GAPDH for the whole-cell group from the respective ion channel CT for each individual cell (see Equation (2)).

ΔCT_Ionchannel_ = CT_Ionchannel_ − [(Mean_Cellgroup_ (CT_TNNT2_) + Mean_Cellgroup_ (CT_GAPDH_))/2] (2)

The five cell groups were compared regarding the percentage of positive cells and the mean normalized expression level in these positive cells (∆CT).

The reliability and robustness of the assay technology were demonstrated (for data, see Supplementary Figure S1b,d). The correlation between our mean single-cell expression data and the bulk-sequencing data (cells were lysed after two weeks in culture) of iCell cardiomyocytes (see Supplementary Figure S1c) shows a negligible influence of singularization, cell selection, and cell age on our results.

### 2.7. Statistics

Note that the single-cell, patch-clamp RT-qPCR method used in this study is technically challenging. Thus, it was not always possible to determine every parameter for every single cell and therefore, *n* may vary between parameters.

SAS software (SAS institute, Cary, NC, USA) was used for the statistical analysis. For every electrophysiological and expression parameter, the mean and SD (per cell group) were calculated. GraphPad Prism was used to create bar graphs. Spotfire (TIBCO, Somerville, MA, USA) software was used to create histograms.

Electrophysiological parameters and ΔCT values (see Section 2.5) were compared between cell groups. Each parameter was analyzed separately, using a linear model for repeated measurements (SAS proc mixed). “Cell group” was considered as a fixed effect. The covariance structure was modelled by a compound symmetry (CS) structure and the covariance parameters were estimated using residual (restricted) maximum likelihood (REML). The pairwise comparisons were made using Student’s *t*-test. The *p* values were not adjusted for multiple comparisons.

Electrophysiological parameters and ΔCT values were compared between beating and non-beating iCell cardiomyocytes. Each parameter was analyzed separately. The beat rate was considered as a fixed effect in a linear model for repeated measurements. The covariance structure was modelled by a compound symmetry (CS) structure, possibly different for the two groups. The covariance parameters were estimated using a residual (restricted) maximum likelihood procedure (REML). 

To assess the relation between the single-cell parameters, each parameter was correlated with every other parameter and Pearson´s coefficient was used for quantification (a positive coefficient indicates direct correlation, a negative coefficient indicates inverse relationship, and the absolute value of the coefficient indicates strength of correlation).

## 3. Results

### 3.1. iCell Cardiomyocytes Do Not Represent Primary Cardiomyocyte Subtypes

The majority (83%) of the 55 individual iCell cardiomyocytes tested were beating spontaneously (17% did not beat). We wondered whether the spontaneously beating cells exhibited the characteristics of the primary nodal cardiomyocytes (i.e., differences in ion channel expression and electrophysiology (c.f. [2]) and assessed whether the beating iCell cardiomyocytes differed from the non-beating cells regarding ion channel expression (percentage of positive cells and CT values) and electrophysiology (Table 1).

In contrast to the primary nodal cardiomyocytes that are smaller than the quiescent atrial and ventricular cardiomyocytes, the beating iCell cardiomyocytes showed a significantly higher cell capacitance (an electrophysiological measure for cell surface and thus cell size) compared to the non-beating cells (Table 1). While primary cardiomyocytes of the central sinoatrial node do not express SCN5A transcripts that encode the voltage-dependent Na^+^ channel Na_v_1.5 [9], all the iCell cardiomyocytes showed fast (Na^+^-driven) inward currents. Furthermore, the beating iCell cardiomyocytes exhibited larger fast inward current amplitudes in response to membrane potential depolarization compared to the non-beating cells (Table 1). These fast inward currents were largely inhibited by the specific Na_V_ channel blocker TTX (30 µM; Supplementary Figure S1) and hence were mediated by the TTX-sensitive Na_V_ channels. As the currents remaining after the application of TTX were insensitive to nifedipine (10 µM), the voltage-gated L-Typ Ca^2+^ (Ca_v_) channels did not relevantly contribute to the fast inward currents recorded in these experiments. As all the cells expressed SCN5A transcripts (encoding Na_V_1.5 channels, 55/55, taking CT values lower than 35 as the mRNA expression threshold), we conclude that the fast inward currents are mediated by the Na_v_1.5 cells in these iCell cardiomyocytes. It is noteworthy that the SCN5A expression in the beating and non-beating iCell cardiomyocytes was comparable (Table 1), demonstrating that Na_v_1.5 expression does not enable clear discrimination between those two populations of iCells.

Likewise, the large majority of the iCell cardiomyocytes expressed transcripts of CACNA1C (encoding Ca_V_1.2 channels; 54/55 cells) and KCNH2 (encoding K_V_11.1/hERG channels, 54/55) with no significant differences in expression levels between the beating and the non-beating cells (Table 1). This is fully in line with the fact that CACNA1C and KCNH2 are expressed in all three cardiomyocyte subtypes of the human heart [2]. Moreover, we detected a similar expression of the atrial ion channel transcript KCNA5 (encoding K_V_1.5 channels) in the beating and non-beating iCell cardiomyocytes (Table 1). Interestingly, expression of the established pacemaking-associated ion channel transcripts HCN4, KCNJ2, and KCNJ4 [7,8] was comparable between the beating and the non-beating iCell cardiomyocytes (as indicated by the CT values and the percentage of positive cells in Table 1), but substantially more beating iCells expressed the pacemaking-associated CACNA1G (18% more) and CACNA1D (19% more) compared to the non-beating cells (Table 1). Strikingly, however, the CACNA1D expression level was even higher in the CACNA1D-positive non-beating iCells (as indicated by lower CT values), suggesting an even higher expression of Ca_v_1.3 channels in these cells (Table 1).

Taken together, the comparison of beating and non-beating iCell cardiomyocytes demonstrated that the phenotypic heterogeneity (i.e., beating vs. non-beating) of iCell cardiomyocytes does not relate to electrophysiological properties or the expression of the cardiac ion channel subunits known from primary adult cells.

**Figure 1:**
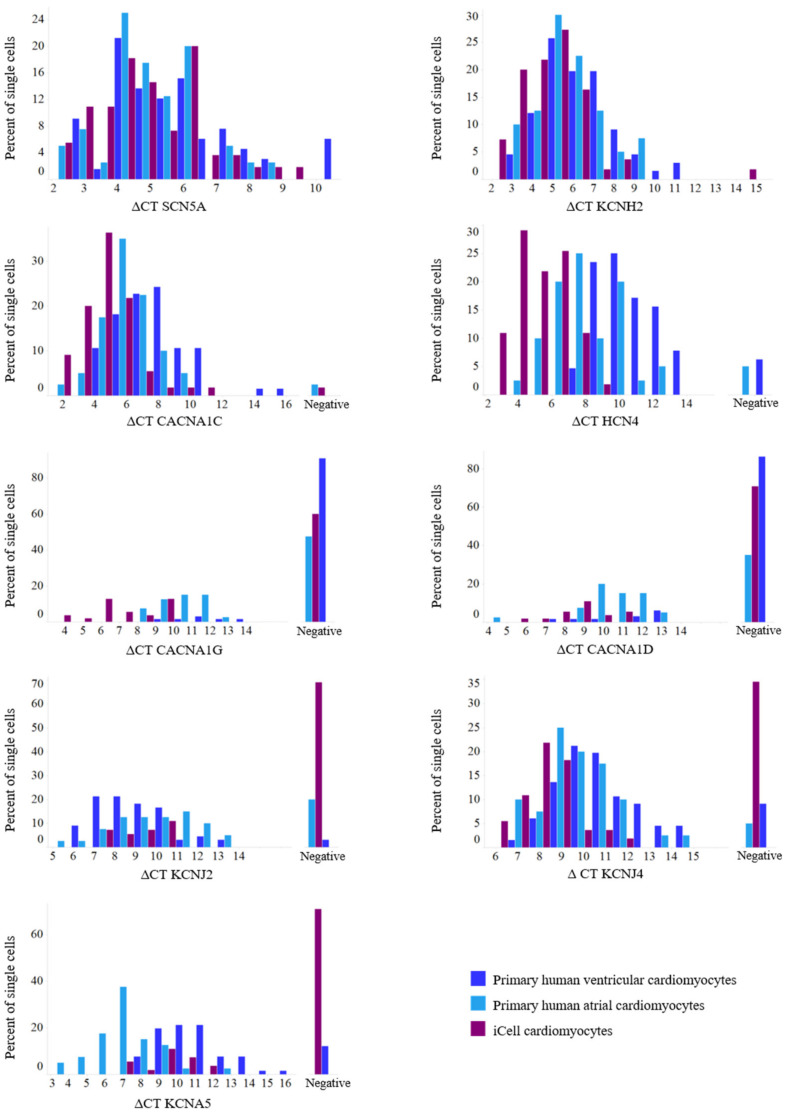
Histograms of expression parameters. Comparisons of distributions of single-cell expression data (ΔCT) between atrial and ventricular human primary cardiomyocytes and iCell cardiomyocytes. For total *n* see Table 3. While for some ion channels (SCN5A, KCNH2, CACNA1C, and KCNJ4) the distributions between primary human atrial and ventricular cardiomyocytes are comparable, the distributions of HCN4, CACNA1D, CACNA1G, and KCNA5 are left-shifted (higher expression) in primary human atrial cardiomyocytes compared to primary human ventricular cardiomyocytes. With respect to KCNJ2, distribution is left-shifted (higher expression) for human primary ventricular cardiomyocytes compared to their atrial counterpart. Comparing distributions between the two primary cardiomyocyte groups and iCell cardiomyocytes shows a shift to the left (higher expression) for the pacemaking-associated ion channels HCN4, CACNA1G, and CACNA1D in iCell cardiomyocytes.

To further investigate the composition of the iCell cardiomyocyte population, we compared the frequency distributions of the single-cell data of the iCell cardiomyocytes with the data of the primary cardiomyocytes (Figure 1).

The expression of SCN5A, KCNH2, and CACNA1C was high and comparable in the primary human atrial and ventricular cardiomyocytes, but the atrial cells showed a higher expression of HCN4, CACNA1G, and CACNA1D (Figure 1; in line with the reports [2]). While the expression of KCNJ2 and KCNJ4 in the atrium and ventricle has been reported inconsistently [6,25], we found comparable expression of KCNJ4 in the primary atrial and ventricular cells and a higher expression of KCNJ2 in the ventricular cardiomyocytes (Figure 1). The expression levels of these genes did not allow for a straightforward transcriptional discrimination of the primary atrial and ventricular cardiomyocytes. In contrast, the frequency distributions of the expression levels of the atrial potassium channel gene KCNA5 [2] revealed two cell populations representing the primary human atrial and ventricular cardiomyocytes (Figure 1). All the primary atrial cardiomyocytes and 88% of the primary ventricular cardiomyocytes expressed KCNA5 transcripts, but the KCNA5 expression levels were clearly higher in the primary atrial cardiomyocytes (mean ΔCT = 7.52) than in the ventricular (mean ΔCT = 10.21). These data demonstrated that KCNA5 is indeed abundant in both cell types in the human heart (as reported previously [25,26]), but due to higher expression is a bona fide marker for human atrial cardiomyocytes.

We then sought to evaluate whether iCell cardiomyocytes represented atrial and/or ventricular CM subtypes utilizing KCNA5 channel transcripts as a cellular marker. The KCNA5 transcripts were expressed in only 29% of all the iCell cardiomyocytes and accordingly were absent in the large majority (71%). Hence, iCell cardiomyocyte cultures indeed consist of at least two different subpopulations, as discriminated by the absence or presence of KCNA5. However, in the respective iCell cardiomyocytes, the expression levels of KCNA5 transcripts (mean ΔCT = 9.70) were significantly lower than in the primary atrial cardiomyocytes of human donors and rather comparable to those detected in the ventricular cardiomyocytes. Beating behavior did not correlate with KCNA5 expression, strongly indicating, rather, that these cells did not represent electrically silent native atrial or ventricular cardiomyocytes.

We conclude that beating iCell cardiomyocytes do not show specific characteristics of nodal cardiomyocytes and that these hiPSC-CMs do not represent distinct primary cardiomyocyte subtypes of the human heart.

### 3.2. A Trend towards Chamber Specificity for hiPSC-Derived Pluricyte Cardiomyocytes

**Figure 2:**
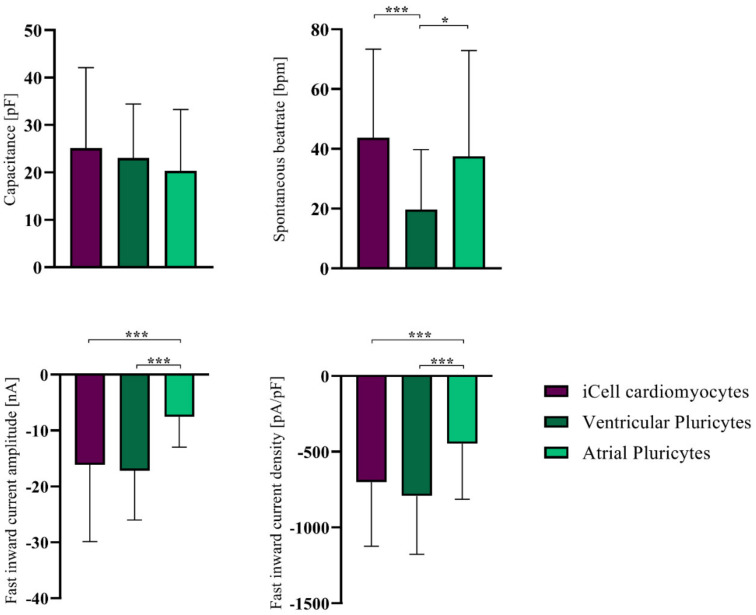
Comparison of electrophysiological parameters between hiPSC-CM groups. Data are shown as mean ± SD. Students *t*-test was used to compare groups. *^,^ *** indicate *p* < 0.05, and *p* < 0.001, respectively. The numbers of total recordings used to calculate means are listed in Table 2.

**Figure 3:**
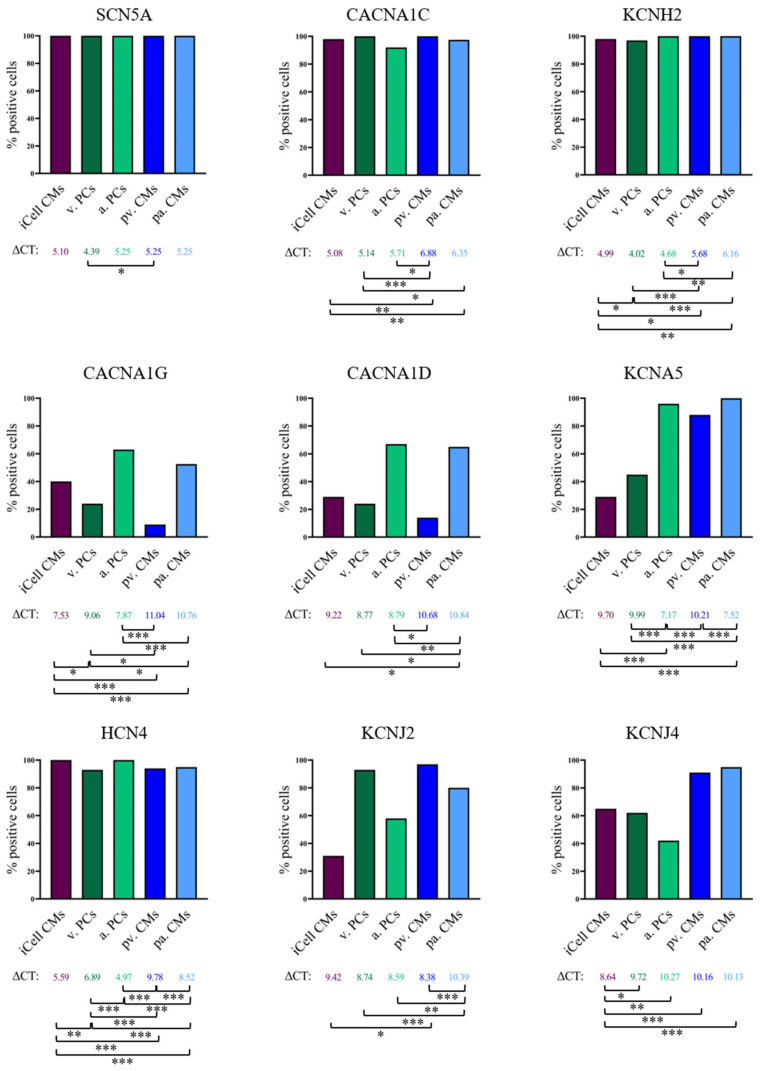
Comparison of cardiomyocyte groups regarding ion channel expression at the single-cell level. Columns represent percentages of positive cells; values shown below the columns are mean ΔCT values (CT values normalized to the mean expression of TNNT2 and GAPDH of the respective cell group) of the positive cells. Students *t*-test was used to compare the ΔCT values between the groups. Significant differences of ΔCT values between groups are indicated with * *p* < 0.05, ** *p* < 0.01 and *** *p* < 0.001. The total numbers used to calculate mean ΔCT values are listed in Table 3. Abbreviations: iCell CMs, iCell cardiomyocytes; v. PCs, ventricular Pluricytes; a. PCs, atrial Pluricytes; pv. CMs, primary ventricular cardiomyocytes; pa. CMs, primary atrial cardiomyocytes.

We then extended our analysis to other commercially available hiPSC-CM models (atrial Pluricytes and ventricular Pluricytes). To evaluate cardiac-chamber-specific characteristics, we again utilized the whole-cell patch-clamp and single-cell RT-qPCR approach to characterize the atrial and ventricular Pluricytes. We first compared their electrophysiological characteristics to the iCell cardiomyocytes (Figure 2, Table 2) and the expression of cardiac ion channel transcripts to the iCell cardiomyocytes and the primary human atrial and ventricular cardiomyocytes (Supplementary Figure S3, Figure 3, and Table 3).

**Table 2:** Cardiomyocyte electrophysiological measurements. Data are presented as means ± standard deviation (SD) and sample size (*n*).

		Capacitance [pF]	Spontaneous Beat Rate [bpm]	Fast Inward Current Amplitude [nA]	Fast Inward Current Density [pA/pF]
iCell CMs	mean	25.15	43.75	−16.12	−700.48
	± SD	16.94	29.60	13.74	423.26
	*n*	72	72	71	71
Ventr. PCs	mean	23.00	19.75	−17.17	−791.36
	± SD	11.44	20.04	8.85	385.25
	*n*	32	32	29	29
Atr. PCs	mean	20.36	37.58	−7.55	−447.24
	± SD	12.91	35.30	5.41	366.18
	*n*	33	33	27	27

**Table 3:** Results of single-cell RT-qPCR. Besides the percentage of positive cells (% pos. cells) and the number of investigated cells (n), also mean (ø) CT values and, mean (ø) ΔCT values are listed. ΔCT values are CT values normalized to the mean expression of TNNT2, and GAPDH of the respective cell group. For details see Methods section. Standard deviations (SD) are identical for CT and ΔCT values because the difference between the two values is a constant.

		HCN4	CACNA1G	CACNA1D	KCNA5	KCNJ4	SCN5A	KCNJ2	CACNA1C	KCNH2	TNNT2	GAPDH
iCell CMs	% pos. cells	100	40	29	29	65	100	31	98	98	100	98
	ø CT	27.94	29.88	31.56	32.05	30.99	27.44	31.77	27.43	27.34	20.38	24.06
	ø ΔCT	5.59	7.53	9.22	9.70	8.64	5.10	9.42	5.08	4.99		
	± SD	1.49	1.89	1.56	1.46	1.29	1.61	1.26	1.81	1.47	1.02	1.17
	n	55	55	55	55	55	55	55	55	55	55	55
Ventr. PCs	% pos. cells	93	24	24	45	62	100	93	100	97	100	100
	ø CT	28.78	30.95	30.66	31.87	31.61	26.27	30.63	27.03	25.91	20.59	23.19
	ø ΔCT	6.89	9.06	8.77	9.99	9.72	4.39	8.74	5.14	4.02		
	± SD	1.19	1.20	1.45	0.85	1.38	1.62	1.98	1.60	1.49	1.11	1.20
	n	29	29	29	29	29	29	29	29	29	29	29
Atr. PCs	% pos. cells	100	63	67	96	42	100	58	92	100	100	100
	ø CT	26.52	29.43	30.34	28.72	31.82	26.80	30.15	27.27	26.23	19.67	23.44
	mean ΔCT	4.97	7.87	8.79	7.17	10.27	5.25	8.59	5.71	4.68		
	± SD	2.19	2.34	2.18	2.72	1.16	1.98	2.07	2.33	2.31	1.81	2.27
	n	24	24	24	24	24	24	24	24	24	24	24
Prim. ventr. CMs	% pos. cells	94	9	14	88	91	100	97	100	100	100	100
	ø CT	28.58	29.84	29.48	29.01	28.95	24.05	27.18	25.68	24.48	16.69	20.91
	ø ΔCT	9.78	11.04	10.68	10.21	10.16	5.25	8.38	6.88	5.68		
	± SD	1.73	1.60	2.19	1.85	1.80	1.93	1.73	2.25	1.80	1.41	1.32
	n	66	66	66	66	66	66	66	66	66	66	66
Prim. atr. CMs	% pos. cells	95	53	65	100	95	100	80	98	100	100	100
	ø CT	28.11	30.35	30.43	27.11	29.72	24.85	29.98	25.95	25.75	17.93	21.25
	ø ΔCT	8.52	10.76	10.84	7.52	10.13	5.25	10.39	6.35	6.16		
	± SD	2.02	1.39	1.84	1.85	1.73	1.44	1.93	1.63	1.67	1.37	1.32
	n	40	40	40	40	40	40	40	40	40	40	40

In contrast to the primary ventricular cardiomyocytes being larger than the primary atrial cardiomyocytes [2], the cell size (as quantified via the electrical capacitance) of the ventricular Pluricytes and the atrial Pluricytes (and iCell cardiomyocytes) was not significantly different. (Figure 2, Table 2). Sixty-six percent and fifty-eight percent of the ventricular Pluricytes and atrial Pluricytes, respectively, were beating spontaneously and accordingly were not quiescent like their primary counterparts. Of note, the ventricular Pluricytes showed significantly slower beating frequencies than the atrial Pluricytes and iCells (Figure 2, Table 2). Hence, we conclude that cell size and beating behavior does not provide any evidence for any apparent chamber specificity of the available Pluricytes cultures.

We then compared the single-cell expression (percentage of positive cells and expression levels) of the cardiac ion channel transcripts to elucidate the potential chamber specificity of the atrial and ventricular Pluricytes on a molecular level (Figure 3 and Table 3). SCN5A, CACNA1C and KCNH2 transcripts were detected in the large majority of primary ventricular/atrial cardiomyocytes (SCN5A: 100%/100%, CACNA1C: 100%/98%, KCNH2: 100%/100%) and ventricular/atrial Pluricytes (SCN5A: 100%/100%, CACNA1C: 100%/92%, KCNH2: 97%/100%), in line with a previous publication reporting I_Na_, I_CaL_, and I_Kr_ in ventricular Pluricytes [18] and a high and comparable expression of these three cardiac ion channel transcripts in the human atrium and ventricle [2,25].

SCN5A levels were highest in the ventricular Pluricytes and were comparable in the other cell groups tested (Figure 3 and Table 3). Indeed, SCN5A is functionally expressed in the atrial and ventricular Pluricytes and iCell cardiomyocytes as all exhibited a voltage-dependent fast inward current that was significantly smaller in mean amplitudes and densities (i.e., current amplitude normalized to cell capacitance) for atrial Pluricytes compared to iCell cardiomyocytes and ventricular Pluricytes (Figure 2, Supplementary Figure S2). Notably, it is well established that primary atrial and ventricular cardiomyocytes [2]) express functional Na_v_ channels.

Based on these results, we conclude that hiPSC-CMs to some extent reproduce expressions of several cardiac ion channel transcripts known from human cardiomyocytes (SCN5A, CACNA1C, and KCNH2). However, as these transcripts are not specific for the different cardiomyocyte subtypes, assessment of these transcripts alone does attribute cardiac chamber specificity to the Pluricytes.

In contrast, the expressions of several other cardiac channel transcripts indicated at least a trend towards some chamber specificity in atrial and ventricular Pluricytes: (i) As suggested previously [2], a higher percentage of primary atrial cardiomyocytes (CACNA1G: 53% and CACNA1D: 65%) and atrial Pluricytes (CACNA1G: 63% and CACNA1D: 67%) were positive for the calcium channel transcripts CACNA1G and CACNA1D than for the ventricular primary cardiomyocytes (CACNA1G: 9% and CACNA1D: 14%) or the ventricular Pluricytes (CACNA1G: 24% and CACNA1D: 24%), respectively (Figure 3 and Table 3). (ii) The atrial primary cardiomyocytes and atrial Pluricytes showed a higher mean HCN4 expression level than their ventricular counterparts (Figure 3 and Table 3). In line with this, HCN4 was reported to be expressed at higher levels in the primary atrial compared to the ventricular cardiomyocytes [2]. (iii) The expression of KCNJ2 was higher in the primary ventricular cardiomyocytes and ventricular Pluricytes compared to their respective atrial counterparts (Figure 3 and Table 3). (iv) KCNJ4 transcripts were detected at similar levels in the primary cardiomyocytes and Pluricytes (Figure 3 and Table 3). However, the percentage of positive cells in the hiPSC-CMs (iCell cardiomyocytes: 65%, ventricular Pluricytes: 62%) was lower than in the primary cardiomyocytes (primary ventricular cells: 91%, primary atrial cells: 95%), with the lowest number of positive cells in the atrial Pluricytes (42%). (v) Importantly, KCNA5—an established marker of primary atrial cardiomyocytes—showed the highest expression levels in the atrial primary cardiomyocytes (see above, Figure 3 and Table 3: 100% positive cells, mean ΔCT = 7.52) and the atrial Pluricytes (96% positive cells, mean ΔCT = 7.17).

Taken together, the expression levels of the distinct ion channel marker genes were clearly related between the atrial and ventricular Pluricytes and their primary human CM counterparts, which indicated a trend towards the chamber specificity of these hiPSC-CM models, as evident on a molecular level.

### 3.3. Single-Cell Correlations between Ion Channel Expression and Electrophysiological Parameters

Ventricular hiPSC-CMs are of special interest for pharmacological safety testing as they may provide a suitable model for pro-arrhythmic risk evaluation (i.e., estimating the risk for the ventricular TdP arrythmia). In this respect, drug-induced prolongation of the action potential can be used as a surrogate marker for assessing the pro-arrhythmic risk of research compounds [27]. As our analyses revealed that ventricular Pluricytes may to some extent reproduce the molecular physiology of human ventricular cardiomyocytes, we reasoned that they might indeed constitute an adequate model to predict the pro-arrhythmic risk of drugs and research substances. To provide a high understanding for this cellular model, we combined action potential recordings with our single-cell approach. For ventricular Pluricytes, the mean upstroke velocity of the APs was of 403.75 ± 150.00 V/s (*n* = 30) and the mean action potential duration at 90% repolarization (APD90) of 271.36 ± 268.50 ms (*n* = 32). These APD90 values were very similar to those known from primary ventricular cardiomyocytes [2]. However, we observed very long action potentials in several ventricular Pluricytes (the maximum APD90 measured was 985.72 ms, 21% of action potentials with APD90 > 500 ms) contributing to a very high variation in the action potential duration of single ventricular Pluricytes. We then sought to identify the ion channels potentially responsible for this heterogeneity and this unusual duration of action potentials in ventricular Pluricytes. However, for the ventricular Pluricytes, the APD90 values did not correlate with the expression in the very same cell of CACNA1C (encoding Ca_V_1.2/I_CaL_) and/or KCNH2 (encoding K_v_11.1/hERG/I_Kr_), both known to contribute to the duration of cardiac action potentials (Supplementary Table S3).

**Figure 4:**
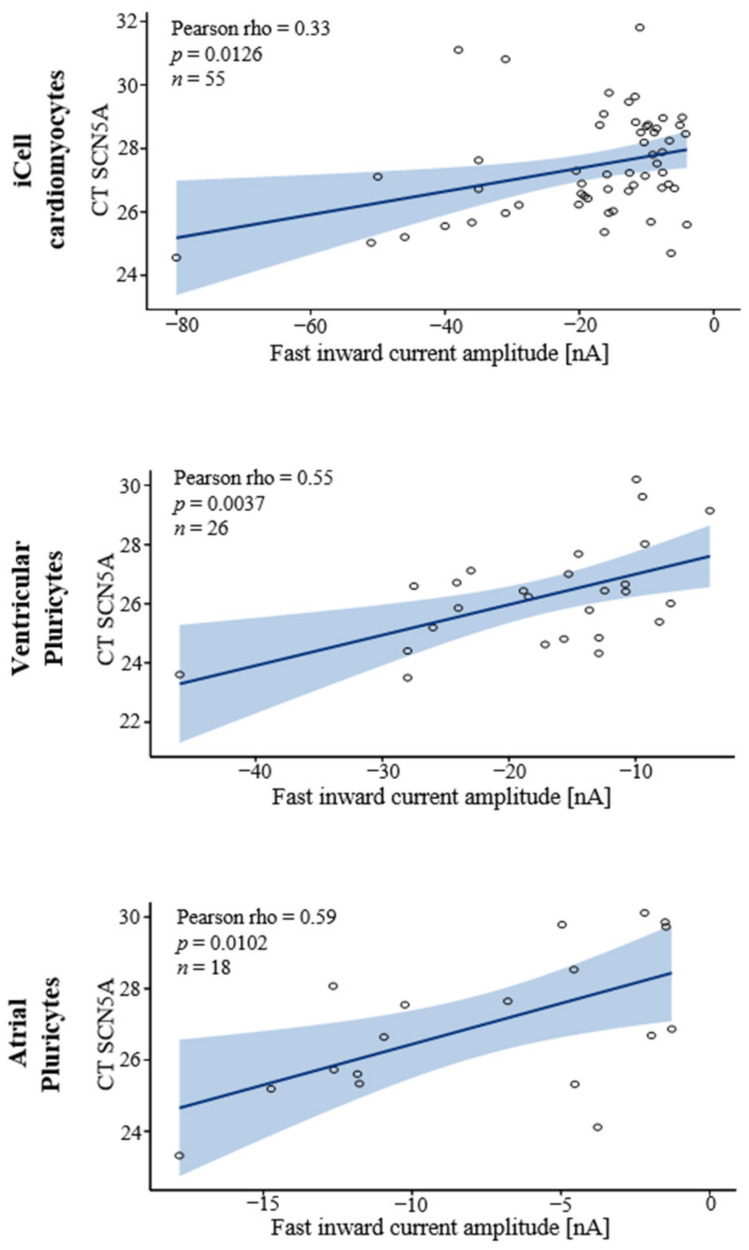
Correlation of SCNA5 expression with the fast inward current. For all three hiPSC-CM groups, CT of SCN5A correlates significantly with the fast inward current amplitude. Blue shaded area indicates 95% confidence limits.

Besides these correlations aiming to explain APD variability in ventricular Pluricytes, we also correlated the other parameters recorded with each other for all three hiPSC-CM groups investigated. To provide a comprehensive view of the complex data set, Supplementary Tables S2–S6 list the results (Pearson´s correlation coefficients r and *p* values) of all the Pearson correlations performed. Interestingly, we did not detect any correlation between the spontaneous beating frequency and the expression of channels relevant for pacemaking (e.g., HCN4, KCNJ2, or KCNJ4) [7,8] in ventricular Pluricytes or the other two hiPSC-CM groups (Supplementary Tables S2–S4). These results indicated that the intrinsically very high variability in the APD of ventricular Pluricytes and the spontaneous beat rate of all the investigated hiPSC-CM groups cannot be explained by transcriptomic inter-cell differences regarding the channels naturally associated with action potential duration and pacemaking. This missing understanding complicates their use. In contrast, the expression of SCN5A correlated positively and significantly with the amplitudes of the fast Na^+^-driven inward currents for all three hiPSC-CM groups (Figure 4, Supplementary Tables S2–S4). Interestingly, for ventricular Pluricytes, an inverse correlation for the fast inward current amplitude and density with the upstroke velocity of the action potential was found (Supplementary Table S3). Moreover, we detected several correlations between the beat rate and the cardiac sodium channel transcript or current. For the ventricular Pluricytes, upstroke velocity correlated positively with beat rate (Supplementary Table S3). Furthermore, for the iCell cardiomyocytes, beat rate and fast inward current amplitude correlated inversely (Supplementary Table S2). Regarding the atrial Pluricytes, the beat rate correlated inversely with the CT of SCN5A (Supplementary Table S4). These data suggest a relationship between beat rate and the cardiac sodium current for hiPSC-CMs, which needs to be fully understood for the interpretation of hiPSC-CM data.

## 4. Discussion

Utilizing a single-cell patch-clamp RT-qPCR approach, our data show that the three commercially available hiPSC-CM cultures differed with respect to electrophysiological parameters and ion channel expression. Whereas the atrial/ventricular Pluricytes exhibit a trend towards chamber specificity, the majority of individual cells of all three hiPSC-CM groups investigated do not represent chamber-specific cell populations present in the adult human heart as they show unusual combinations of the investigated parameters.

Beyond doubt, subtype-specific hiPSC-CMs are promising tools for diverse areas of biomedical research and drug development. Theoretically, one method to get access to such subtype-specific hiPSC-CMs is the enrichment or separation of a specific subtype from potentially heterogeneous populations of non-directed cardiac differentiation approaches [10]. To apply this method successfully, knowledge of the cellular composition and an abundance of nodal, atrial, and ventricular cells in the culture is pivotal. Of note, the cellular composition of such cultures has been analyzed and debated rather controversially in the recent literature, based on electrophysiological or transcriptomic evidence. Whereas some publications reported action potential morphologies in hiPSC-CM populations reminiscent of nodal, atrial, or ventricular(-like) cells [14,15,28], other studies provided evidence for multiple or even a broad spectrum of action potential phenotypes in individual hiPSC-CMs [29,30,31,32], thereby refuting to some extent the abundance of three distinct cardiomyocyte subtypes. The majority of single-cell RNA-sequencing studies did not detect distinct cardiac subtype-related clusters in hiPSC-CMs [19,33,34]. However, one study [35] reported expression related to atrial and ventricular cardiomyocytes. Our study improved our understanding of non-directed hiPSC-CM populations by providing analysis of the combination of electrophysiological characteristics and gene expression in single iCell cardiomyocytes. Taken together, beating iCell cardiomyocytes do not show specific characteristics associated with nodal cardiomyocytes (apart from macroscopic beating behavior) and—with respect to the identified unusual combinations of cardiac ion channel transcripts—the iCell cardiomyocyte population does not consist of and does not represent different adult primary cardiomyocyte subtypes.

Besides iCell cardiomyocytes, we assessed atrial and ventricular Pluricytes. Our results deciphered several characteristics of primary subtype-specificity in atrial and ventricular Pluricytes (e.g., with respect to the expression of KCNA5, CACNA1G, CACNA1D, HCN4, and KCNJ2). As these characteristics indicated a trend towards the chamber specificity of atrial and ventricular Pluricytes, appropriate differentiation approaches seem to bear the potential to derive more subtype-specific hiPSC-CM models. In addition to the mentioned subtype-associated parameters, we also evaluated parameters (the expression of SCN5A, CACNA1C, and KCNH2) associated with physiologically important cardiac ion currents (cardiac sodium, calcium, and HERG current, respectively) to further validate hiPSC-CMs as a model for primary cardiomyocytes in research or drug discovery. The robust expression of these channels and the presence of the sodium-driven fast inward current in all hiPSC-CMs confirms the cardiac-identity of hiPSC-CMs and potentially offers an experimental platform to study those ion channels in a close-to cardiomyocyte environment.

In contrast to their quiescent primary counterparts, the ventricular and atrial Pluricytes (and iCell cardiomyocytes) were beating spontaneously, which clearly highlights the significant differences between these hiPSC-CMs and primary human cardiomyocytes. Consistent with that, the three hiPSC-CM groups in our study showed a higher mean expression of the pacemaking-related and nodal-associated cardiac ion channel transcripts HCN4, CACNA1D, and CACNA1G [4,5,7] than the primary atrial and ventricular cardiomyocytes. Strikingly, our results showed that almost every cardiomyocyte investigated was positive for HCN4 completely independently of the beating behavior. Indeed, the presence of HCN4 in the hiPSC-CMs was also reported previously on protein (in 90–92% of the single hiPSC-CMs investigated [36]) and on a functional level (I_f_ current in iCell cardiomyocytes [15]). Although HCN4 is strongly associated with nodal cells, the transcript was also detected in a high percentage of human primary atrial and ventricular cardiomyocytes, however at lower expression levels, as in the hiPSC-CMs. Together with the results of previous publications (recording of I_f_ current in primary ventricular cardiomyocytes [37]), these data rather indicate that HCN4 is not specifically expressed in nodal cardiomyocytes.

The strong nodal-related component in hiPSC-CMs also becomes evident in the phenotype of single cells (i.e., the combination of electrophysiological and transcriptomic attributes in single cells). In line with a previous report [38], every hiPSC-CM investigated in our study expressed sodium-driven fast inward currents and SCN5A transcripts, and indeed, the SCN5A expression levels correlated with the fast inward current amplitude. Thus, the spontaneously beating iCell cardiomyocytes (83%) and Pluricytes (ventricular: 66%, atrial: 58%) also demonstrated a prominent fast inward current mainly driven by I_Na_ and were positive for SCN5A. It is widely accepted that, while spontaneous generation of action potentials is a clearly nodal attribute, the presence of a prominent I_Na_-driven fast inward current and a high expression of SCN5A are non-nodal characteristics (also associated with adult atrial and ventricular primary cardiomyocytes [2]). Although data on the presence of I_Na_ in the human central sinoatrial node are rare, a previous report found a large inward current with characteristics of I_Na_ in two out of a total of three human sinoatrial node cells that were evaluated with recording conditions similar to the ones we used in the current study [39]. Considering, however, that the expression of SCN5A is restricted to the peripheral sinoatrial node [9], the two cells with the I_Na_-like current might be representative of the peripheral region of the sinoatrial node and not of its central region. Thus, our single-cell data further indicated that the majority of hiPSC-CMs combine attributes of multiple adult cardiomyocyte types. A functional relation between the spontaneous beat rate and the sodium current was reported for human embryonic stem-cell-derived cardiomyocytes in a previous study [40]. In line with this, we find an association between beat rate and sodium current in our data. The beat rate of the iCell cardiomyocytes was inversely correlated with the fast inward current amplitude (which correlates with expression of SCN5A), and the beat rate of the atrial Pluricytes significantly correlated with the SCN5A expression (that in turn correlates with the fast inward current amplitude). Moreover, the beat rate of the ventricular Pluricytes correlated positively with the upstroke velocity of the action potential. In addition, the beating iCell cardiomyocytes showed a significantly larger fast inward current amplitude than the non-beating cells. These results indicate a role of the cardiac sodium current in the generation of the spontaneous beating of hiPSC-CMs, a mechanism not expected in primary cardiomyocytes of the central sinoatrial node.

The results of our study highlight several advantages but also outline the limitations of hiPSC-CMs as model systems in research and drug discovery. The cardiac ion channel transcripts SCN5A, CACNA1C, and especially KCNH2 are infamous for their contribution to genetic cardiac disease and/or cardiovascular side effects [41,42]. Robust expression of these channels in the three investigated hiPSC-CM models enables analysis of the respective currents in these cells in an experimental setting apparently closer to the native environment than most recombinant expression systems. Similarly, atrial Pluricytes express KCNA5 at levels comparable to primary atrial cardiomyocytes and may thus be used to screen for substances that bind to or modulate the atrial-associated ion channel K_V_1.5 (KCNA5), a target proposed for the treatment of atrial fibrillation [43]. However, we want to point out that the selective analysis of a respective ion current (e.g., with a whole cell patch clamp) may be hampered by the expression of several cardiac ion channels in the same hiPSC-CM. Thus, pharmacological intervention may be needed to isolate the current of interest.

Along these lines, hiPSC-CMs may offer a straightforward evaluation of the arrhythmogenic risk of drug candidates through the quantification of drug-induced elongation of the action potential, which can be performed with a whole cell patch clamp or with non-invasive methods using voltage-sensitive fluorescent dyes [44,45]. Notably, the estimation of the TdP risk of drug candidates necessitates ideally ventricular cardiomyocytes. Due to their potential chamber specificity, ventricular Pluricytes appear promising in this respect, but the intrinsic variability of their APD was extremely high (action potentials were substantially longer in several ventricular Pluricytes than in primary ventricular cardiomyocytes), and action potential duration did not correlate with the expression of ion channels known to determine APD (SCN5A, CACNA1C, and KCNH2). Thus, variability of APD cannot be reasonably explained by the expression of cardiac ion channels on the transcriptomic level at present, which also limits applicability of this cell model in drug discovery. There are numerous plausible explanations for this lack of correlation. For example, the duration of an action potential is influenced by many other ionic currents than just I_Kr_ and I_CaL_, and transcripts of just these two ion channels may not be sufficient to explain APD90. Moreover, ion channels are known to be regulated by different complex mechanisms after translation, indicating that regulation of APD may not be simplified by assessment of the transcription of two ion channel subunits. Independently, this heterogeneity may cause problems when using the cells for detection of drug-induced changes in APD in a low-throughput assay (usually resulting in small sample sizes), such as the manual patch-clamp technique. In this case, the selection criteria (use of cells within a defined APD range) or the increase in sample size using a higher throughput platform could help to reliably detect changes in action potential duration despite the high basal heterogeneity.

Likewise, the spontaneous beating of hiPSC-CMs may enable their application to the study of the potential chronotropic effects of drug candidates. However, a previous study showed that the spontaneous beating of hiPSC-CMs rather complicates the interpretation of pharmacological safety investigations [46]. We found that the beating behavior of hiPSC-CM does not correlate with the expression of the known determinants of spontaneous activity (e.g., HCN4, KCNJ2, or KCNJ4).

Both APD and spontaneous beat rate are complex parameters that depend on the integration of multiple ionic currents. As our study showed that the majority of individual hiPSC-CMs expressed a combination of ionic channels that are not expected to be present in adult primary cardiomyocyte subtypes, such complex parameters might be driven by a set of ion currents different from the expected physiological one (e.g., possible contribution of the cardiac sodium current to spontaneous beat rate). Numerous publications used reference compounds (specifically targeting one ion current) to show that the well-known ion currents contribute to APD and beat rate (e.g., field potential prolongation due to hERG-blockage with E4031 and the slowing of spontaneous beating due to I_f_-blockage with ivabradine [45,47]). However, in contrast to such reference compounds, interpretation of the results from drug candidates is complicated by the fact that these compounds are often unselective multi-ion-channel blockers with unknown targets. According to our results, an ion channel panel spanning not only ventricular channels needs to be kept in mind for cautious interpretation of such drug-induced changes, and the usage of human primary cardiomyocytes might be a preferred option.

These examples highlight that the applicability of hiPSC-CMs strongly depends on the intended application and aim. Besides the above-mentioned applications, hiPSC-CMs are under investigation for their suitability for evaluating other safety issues. For example, they were shown to be useful for the assessment of anthracycline-induced cardiotoxicity [48,49]. We propose that the utilization of hiPSC-CM models for a specific application has to be complemented with a characterization and validation tailored to this specific application.

## Figures and Tables

**Table 1:** Comparison of spontaneously beating and non-beating iCell cardiomyocytes.

		iCell Cardiomyocytes	
Spontaneous Beat Rate (*n* Cells)		=0 (8)	>0 (47)
HCN4	% pos. cells ø CT ± SD	100 27.8 ± 0.6	100 28.0 ± 0.2
CACNA1G	% pos. cells ø CT ± SD	25 30.1 ± 0.1	43 29.9 ± 0.4
CACNA1D	% pos. cells ø CT ± SD	13 29.5 ± 0.03	32 31.7 ± 0.4 ***
KCNA5	% pos. cells ø CT ± SD	38 32.6 ± 0.4	28 31.9 ± 0.4
KCNJ4	% pos. cells ø CT ± SD	75 30.8 ± 0.6	64 31.0 ± 0.2
SCN5A	% pos. cells ø CT ± SD	100 27.9 ± 0.4	100 27.4 ± 0.2
KCNJ2	% pos. cells ø CT ± SD	25 31.6 ± 1.2	32 31.8 ± 0.3
CACNA1C	% pos. cells ø CT ± SD	100 27.7 ± 0.9	98 27.4 ± 0.3
KCNH2	% pos. cells ø CT ± SD	100 27.9 ± 0.7	98 27.2 ± 0.2
ø Capacitance [pF] ± SD		14.68 ± 1.80	27.25 ± 2.28 ***
ø Fast inward current amplitude [nA] ± SD		−10.36 ± 2.33	−17.30 ± 1.87 *
ø Fast inward current density [pA/pF] ± SD		−686.1 ± 125.8	703.4 ± 55.3

For illustrative purposes, an arbitrary threshold of 15% was defined and the differences in percentage of positive cells (% pos. cells) between beating and non-beating hiPSC-CMs > 15% are highlighted with a yellow shading. In the case of a significant difference between beating and non-beating hiPSC-CMs (* *p* < 0.05 and *** *p* < 0.001:), mean (ø) values are printed in blue. The covariance parameters were estimated using residual (restricted) maximum likelihood procedure (REML).

## Data Availability

Data is contained within the article and Supplementary Materials.

## Supplementary Materials

The following are available online at https://www.mdpi.com/article/10.3390/cells10123370/s1, Figure S1: Reliability and reproducibility of experiments, Figure S2: Representative fast inward current traces for iCell cardiomyocytes, ventricular Pluricytes, and atrial Pluricytes, Figure S3: Binary expression of cardiac ion channels in single cardiomyocytes. Table S1: Detailed information on donor hearts. Table S2: Single-cell correlations for iCell cardiomyocytes. Table S3: Single-cell correlations for ventricular Pluricytes. Table S4: Single-cell correlations for atrial Pluricytes. Table S5: Single-cell correlations for primary human ventricular cardiomyocytes. Table S6: Single-cell correlations for primary human atrial cardiomyocytes. Table S7: TaqMan gene expression assays used for experiments.

## Abbreviations

CACNA1C/D/G	calcium voltage-gated channel subunit alpha1 C/D/G
Ca_V_1.2/1.3	voltage-gated L-type calcium channel subunit alpha 1.2/1.3
Ca_V_3.1	voltage-gated T-type calcium channel subunit alpha 3.1
cDNA	complementary deoxyribonucleic acid
CiPA	Comprehensive in vitro Proarrhythmia Assay
DNA	deoxyribonucleic acid
CT	cycle threshold
ΔCT	normalized cycle threshold
D-PBS	Dulbecco´s phosphate buffered saline
EC solution	extracellular solution
GAPDH	glyceraldehyde-3-phosphate dehydrogenase
HCN4	hyperpolarization activated cyclic nucleotide gated potassium channel 4
hERG channel	human ether-a-go-go related gene channel
hiPSC-CM	human induced pluripotent stem cell derived cardiomyocytes
IC solution	intracellular solution
I_CaL_	L-type calcium current
I_CaT_	T-type calcium current
I_f_	funny current
I_K1_	inward rectifier potassium current
I_Kr_	rapid component of the delayed rectifier potassium current
I_Kur_	ultra-rapid delayed rectifier potassium current
I_Na_	cardiac sodium current
KCNA5	potassium voltage-gated channel subfamily A member 5
KCNH2	potassium voltage-gated channel subfamily H member 2
KCNJ2/4	potassium inwardly rectifying channel subfamily J member 2/4
Kir2.1/2.3	inward-rectifier potassium ion channel subunit 2.1/2.3
K_V_ 1.5/1.11	voltage-gated potassium channel subunit 1.5/1.11
mRNA	messenger ribonucleic acid
RNA	ribonucleic acid
RNase	ribonuclease
rt	room temperature
RT	reverse transcription
SCN5A	sodium voltage-gated channel alpha subunit 5
SD	standard deviation
RT-qPCR	reverse transcription quantitative polymerase chain reaction
TdP	Torsade de Pointes
TNNT2	troponin T2, cardiac type
TTX	tetrodotoxin
