# Supplementary material for: Ion Channel Expression and Electrophysiology of Singular Human (Primary and Induced Pluripotent Stem Cell-Derived) Cardiomyocytes
Source: Cells. 2021 Nov 30;10(12):3370. doi: 10.3390/cells10123370 (PMC8699770; doi:10.3390/cells10123370)
Supplement: Supplementary file 1 [file cells-10-03370-s001.zip › cells-1456381-supplementary.pdf]

Supplementary information

Supplementary figures

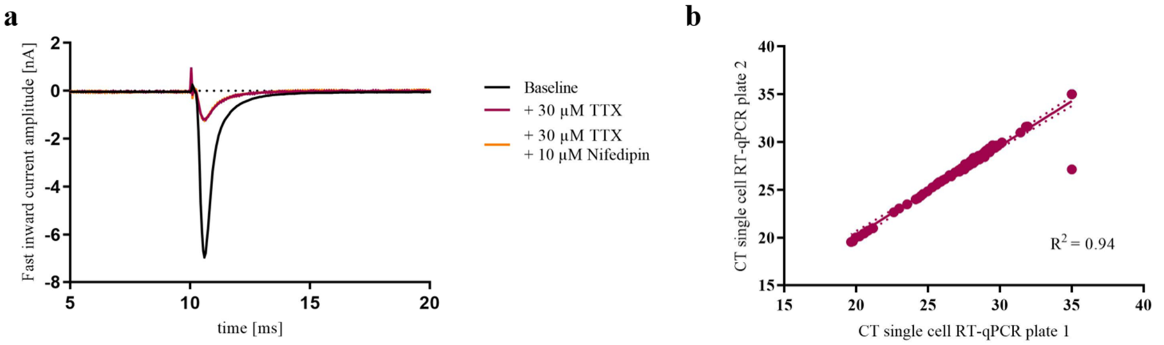

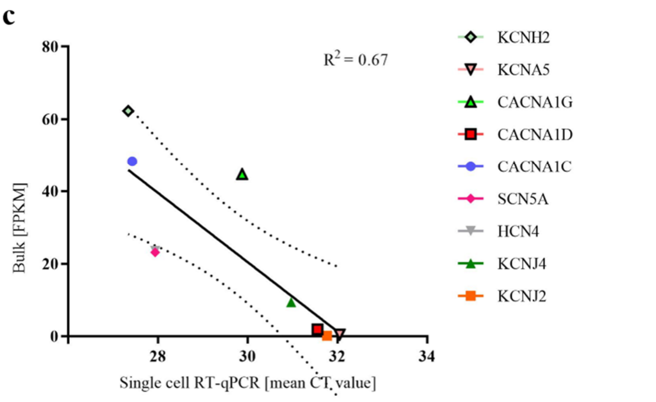

d

		SCN5A			CACNA1C		
		CT Rep. 1	CT Rep. 2	ø CT	CT Rep. 1	CT Rep. 2	ø CT
**iCell 1**	20x	28.8	29.0	28.9	30.1	30.1	30.1
	15x	28.2	28.2	28.2	29.5	29.3	29.4
	10x	27.6	27.6	27.6	28.9	28.8	28.9
**iCell 2**	20x	30.0	30.2	30.1	32.0	31.9	31.9
	15x	29.5	29.7	29.6	31.4	31.3	31.3
	10x	28.8	28.8	28.8	30.6	30.8	30.7

Supplementary Figure S1: Reliability and reproducibility of experiments**.** All depicted validations have been performed using iCell cardiomyocytes. **(a)** Representative trace of the fast inward current. The current can be blocked with TTX, indicating the cardiac sodium channel Nav1.5 (SCN5A) mainly conducts it. **(b)** The qPCR for different targets (HCN4, SCN5A, CACNA1C, KCNH2, TNNT2 and GAPDH) of 12 iCell cardiomyocytes was run twice on two different plates. CT values of both plates for each cell and target were plotted against each other. High correlation indicates low inter-plate variability. **(c)** Correlation of results of bulk sequencing (data were taken from [19]) and mean CT values of iCell cardiomyocytes. **(d)** Representative raw data results of qPCR of two iCell cardiomyocytes (iCell 1 and iCell 2) for SCN5A and CACNA1C. Note that dilutions of pre-amplified cDNA (10 – 15 -20x) reflect CT values (difference between 20x and 10x is one CT value) and that CT values of technical replicates are highly reproducible

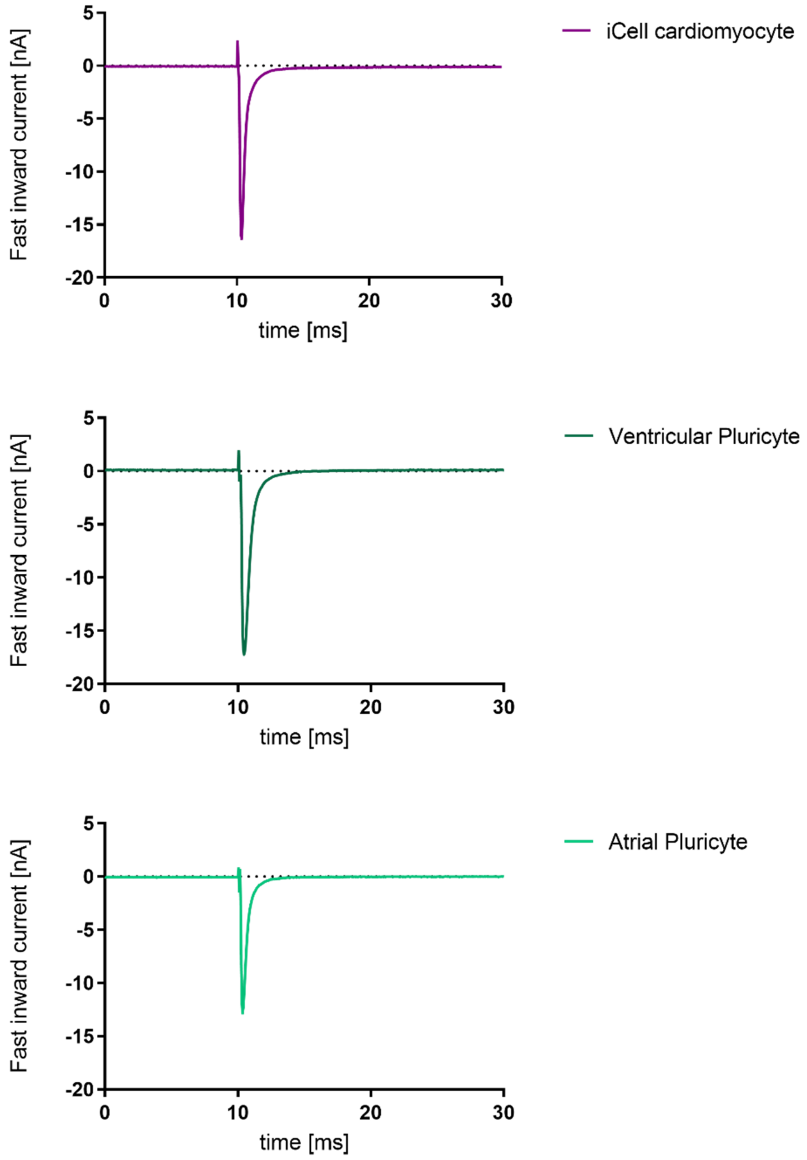

Supplementary Figure S2: Representative fast inward current traces for iCell cardiomyocytes, ventricular Pluricytes, and atrial Pluricytes. To record the fast inward current, cells were clamped at a holding potential of -100 mV (to make sure sodium channels were not inactivated) followed by a voltage step to -30 mV for 100 ms. The numbers of total recordings are listed in Table 4.

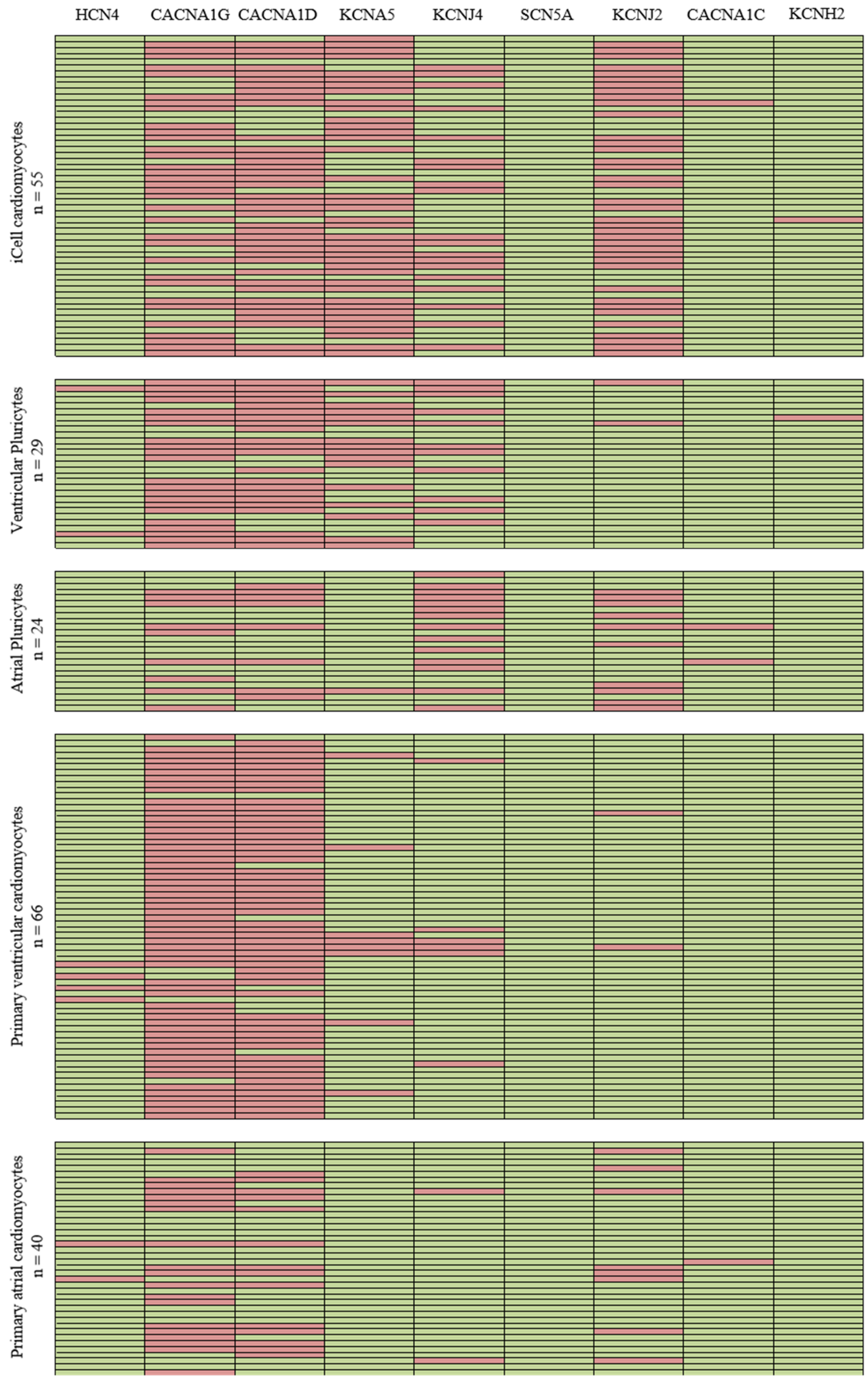

Supplementary Figure S3: Binary expression of cardiac ion channels in single cardiomyocytes. Each row represents one cell. A green/red box indicates that the cell was positive/negative (for details see Methods section) for the respective ion channel.

Supplementary tables

Supplementary Table S1: Detailed information on donor hearts. In total four hearts were used. The number of cells per heart, tested in the current study, are listed. Abbreviations: F, Female; M, Male; BMI, Body Mass Index; COD, Cause Of Death; EF, Ejection Fraction; CVA, Cerebrovascular Accident; ICH, Intracranial Haemorrhage; [a]Organ procurement organization could not transplant the heart and consequently no echocardiography was performed; N/A, Not available.

Heart#	# of atrial cells	# of ventr. cells	Age	Sex	Ethnicity	BMI	COD	EF (%)
1	20	44	44	F	Hispanic	23.9	Anoxia	70
2	-	20	50	F	Caucasian	27.0	CVA/ICH/Stroke	70
3	1	26	48	M	Hispanic	29.7	CVA/ICH/Stroke	73
5	19	-	48	M	Caucasian	25.0	Head Trauma/Blunt Injury	N/A[a]

Supplementary Table S2: Single-cell correlations for iCell cardiomyocytes. For every pairwise correlation, Pearson´s correlation coefficient r, the p-value and *n* is indicated. For expression parameters (CT), only positive cells are included in the analysis. Significant correlations (p < 0.05) are highlighted.

Pearson Correlation Coefficients
Prob > |r| under H0: Rho=0
Number of Observations

	Capacitance [pF]	CT TNNT2	CT GAPDH	Beat rate [bpm]	Fast inward current amplitude [nA]	Fast inward current density [nA/pF]	CT KCNJ4	CT HCN4	CT SCN5A	CT CACNA1C	CT KCNH2	CT CACNA1D	CT CACNA1G	CT KCNA5	CT KCNJ2
Capacitance [pF]															
CT TNNT2	-0.47948<br>0.0002<br>55														
CT GAPDH	-0.41380<br>0.0019<br>54	0.63821<br><.0001<br>54													
Beat rate [bpm]	0.39615<br>0.0006<br>72	-0.20614<br>0.1311<br>55	-0.09661<br>0.4871<br>54												
Fast inward current amplitude [nA]	-0.52661<br><.0001<br>71	0.25793<br>0.0573<br>55	0.40036<br>0.0027<br>54	-0.32099<br>0.0063<br>71											
Fast inward current density [nA/pF]	0.22838<br>0.0554<br>71	-0.18581<br>0.1744<br>55	0.07108<br>0.6095<br>54	-0.04794<br>0.6914<br>71	0.57352<br><.0001<br>71										
CT KCNJ4	-0.14583<br>0.3961<br>36	0.25792<br>0.1288<br>36	0.33011<br>0.0528<br>35	-0.03112<br>0.8570<br>36	0.06821<br>0.6926<br>36	-0.03945<br>0.8193<br>36									
CT HCN4	-0.13913<br>0.3110<br>55	0.57387<br><.0001<br>55	0.62395<br><.0001<br>54	-0.12547<br>0.3614<br>55	0.11026<br>0.4229<br>55	0.01460<br>0.9157<br>55	0.46450<br>0.0043<br>36								
CT SCN5A	-0.31477<br>0.0193<br>55	0.60979<br><.0001<br>55	0.57934<br><.0001<br>54	-0.22416<br>0.0999<br>55	0.33424<br>0.0126<br>55	0.12547<br>0.3614<br>55	0.28567<br>0.0912<br>36	0.63050<br><.0001<br>55							
CT CACNA1C	-0.15034<br>0.2779<br>54	0.65659<br><.0001<br>54	0.54573<br><.0001<br>53	-0.05731<br>0.6806<br>54	0.13894<br>0.3164<br>54	-0.03184<br>0.8192<br>54	0.48559<br>0.0031<br>35	0.77101<br><.0001<br>54	0.63570<br><.0001<br>54						
CT KCNH2	-0.33075<br>0.0146<br>54	0.66434<br><.0001<br>54	0.67170<br><.0001<br>53	-0.27414<br>0.0449<br>54	0.24597<br>0.0730<br>54	-0.04697<br>0.7359<br>54	0.54151<br>0.0008<br>35	0.74212<br><.0001<br>54	0.63704<br><.0001<br>54	0.63982<br><.0001<br>53					
CT CACNA1D	-0.51714<br>0.0402<br>16	0.21706<br>0.4194<br>16	0.35693<br>0.1747<br>16	-0.39439<br>0.1306<br>16	0.12324<br>0.6493<br>16	-0.30098<br>0.2573<br>16	0.26174<br>0.4112<br>12	0.38780<br>0.1378<br>16	0.27524<br>0.3022<br>16	0.20048<br>0.4566<br>16	0.45558<br>0.0879<br>15				
CT CACNA1G	-0.62623<br>0.0018<br>22	0.26047<br>0.2417<br>22	0.50993<br>0.0153<br>22	-0.52551<br>0.0120<br>22	0.30266<br>0.1710<br>22	-0.09500<br>0.6741<br>22	0.35683<br>0.1597<br>17	0.34438<br>0.1165<br>22	0.42360<br>0.0495<br>22	0.33256<br>0.1305<br>22	0.46296<br>0.0300<br>22	0.72164<br>0.0433<br>8			
CT KCNA5	0.20682<br>0.4422<br>16	0.43548<br>0.0918<br>16	0.24729<br>0.3558<br>16	-0.04316<br>0.8739<br>16	0.07185<br>0.7915<br>16	0.21550<br>0.4228<br>16	0.08566<br>0.8140<br>10	0.35294<br>0.1800<br>16	0.34730<br>0.1875<br>16	0.51606<br>0.0407<br>16	0.40845<br>0.1163<br>16	-0.75064<br>0.0519<br>7	-0.23409<br>0.6134<br>7		
CT KCNJ2	0.28982<br>0.2592<br>17	0.24953<br>0.3341<br>17	0.35331<br>0.1642<br>17	0.15514<br>0.5521<br>17	0.09899<br>0.7054<br>17	0.60831<br>0.0096<br>17	0.42002<br>0.1349<br>14	0.43623<br>0.0800<br>17	0.52937<br>0.0289<br>17	0.28982<br>0.2592<br>17	0.35981<br>0.1560<br>17	-0.09204<br>0.8004<br>10	-0.00349<br>0.9935<br>8	0.75423<br>0.0501<br>7	

Supplementary Table S3: Single-cell correlations for ventricular Pluricytes. For every pairwise correlation, Pearson´s correlation coefficient r, the p-value and *n* is indicated. For expression parameters (CT), only positive cells are included in the analysis. Significant correlations ($p < 0.05$) are highlighted.

Pearson Correlation Coefficients<br>Prob > |r| under H0: Rho=0<br>Number of Observations	Capacitance [pF]	CT TNNT2	CT GAPDH	Beat rate [bpm]	Fast inward current amplitude [nA]	Fast inward current density [nA/pF]	Upstroke velocity [V/ms]	APD90 [ms]
Capacitance [pF]								
CT TNNT2	-0.47201<br>0.0097<br>29							
CT GAPDH	-0.56797<br>0.0013<br>29	0.72151<br><.0001<br>29						
Beat rate [bpm]	0.25750<br>0.1548<br>32	0.22268<br>0.2456<br>29	0.14911<br>0.4401<br>29					
Fast inward current amplitude [nA]	-0.55135<br>0.0019<br>29	0.33893<br>0.0903<br>26	0.25595<br>0.2069<br>26	-0.28312<br>0.1367<br>29				
Fast inward current density [nA/pF]	0.38774<br>0.0377<br>29	-0.24292<br>0.2318<br>26	-0.40638<br>0.0394<br>26	-0.09831<br>0.6119<br>29	0.48312<br>0.0079<br>29			
Upstroke velocity [V/ms]	0.32201<br>0.0827<br>30	0.09515<br>0.6369<br>27	-0.05456<br>0.7869<br>27	0.44634<br>0.0134<br>30	-0.54228<br>0.0024<br>29	-0.39389<br>0.0345<br>29		
APD90 [ms]	0.14464<br>0.4296<br>32	-0.32318<br>0.0873<br>29	-0.33659<br>0.0742<br>29	-0.08132<br>0.6582<br>32	0.03141<br>0.8715<br>29	0.20910<br>0.2763<br>29	0.19974<br>0.2900<br>30	
CT KCNJ4	0.25169<br>0.3137<br>18	0.35900<br>0.1434<br>18	0.26974<br>0.2790<br>18	-0.17098<br>0.4975<br>18	0.18620<br>0.4899<br>16	0.30188<br>0.2558<br>16	-0.13674<br>0.6008<br>17	0.09025<br>0.7217<br>18
CT HCN4	-0.58489<br>0.0014<br>27	0.54664<br>0.0032<br>27	0.80464<br><.0001<br>27	-0.09305<br>0.6443<br>27	0.28815<br>0.1721<br>24	-0.38461<br>0.0635<br>24	-0.08920<br>0.6716<br>25	-0.31381<br>0.1109<br>27
CT SCN5A	-0.54420<br>0.0023<br>29	0.73648<br><.0001<br>29	0.66461<br><.0001<br>29	-0.14862<br>0.4416<br>29	0.54891<br>0.0037<br>26	-0.04690<br>0.8200<br>26	-0.29704<br>0.1324<br>27	-0.17614<br>0.3607<br>29
CT CACNA1C	-0.26417<br>0.1661<br>29	0.70566<br><.0001<br>29	0.78418<br><.0001<br>29	0.17565<br>0.3621<br>29	0.11549<br>0.5742<br>26	-0.29148<br>0.1485<br>26	0.24990<br>0.2087<br>27	-0.28120<br>0.1395<br>29
CT KCNH2	-0.50268<br>0.0064<br>28	0.83824<br><.0001<br>28	0.77068<br><.0001<br>28	0.18654<br>0.3419<br>28	0.41888<br>0.0372<br>25	-0.07150<br>0.7341<br>25	-0.09468<br>0.6455<br>26	-0.28008<br>0.1489<br>28
CT CACNA1D	-0.60008<br>0.1543<br>7	0.13146<br>0.7787<br>7	0.50153<br>0.2515<br>7	-0.16058<br>0.7309<br>7	0.42476<br>0.3422<br>7	-0.00830<br>0.9859<br>7	-0.45459<br>0.3055<br>7	0.04534<br>0.9231<br>7
CT CACNA1G	-0.61184<br>0.1443<br>7	0.20023<br>0.6668<br>7	0.33206<br>0.4668<br>7	-0.26131<br>0.5714<br>7	0.89202<br>0.0069<br>7	0.47738<br>0.2787<br>7	-0.23095<br>0.6183<br>7	-0.62289<br>0.1351<br>7
CT KCNA5	0.05814<br>0.8504<br>13	0.05273<br>0.8642<br>13	0.15099<br>0.6224<br>13	0.38399<br>0.1952<br>13	-0.48551<br>0.0926<br>13	-0.55685<br>0.0481<br>13	0.51997<br>0.0685<br>13	-0.54486<br>0.0542<br>13
CT KCNJ2	-0.15219<br>0.4486<br>27	0.53316<br>0.0042<br>27	0.46542<br>0.0144<br>27	-0.08619<br>0.6691<br>27	0.08833<br>0.6746<br>25	-0.20747<br>0.3197<br>25	-0.09146<br>0.6568<br>26	-0.04127<br>0.8381<br>27

Supplementary Table S3 continued: Single-cell correlations for ventricular Pluricytes**.** For every pairwise correlation, Pearson´s correlation coefficient r, the p-value and *n* is indicated. For expression parameters (CT), only positive cells are included in the analysis. Significant correlations ($p < 0.05$) are highlighted.

Pearson Correlation Coefficients<br>Prob > |r| under H0: Rho=0<br>Number of Observations									
	CT KCNJ4	CT HCN4	CT SCN5A	CT CACNA1C	CT KCNH2	CT CACNA1D	CT CACNA1G	CT KCNA5	CT KCNJ2
Capacitance [pF]									
CT TNNT2									
CT GAPDH									
Beat rate [bpm]									
Fast inward current amplitude [nA]									
Fast inward current density [nA/pF]									
Upstroke velocity [V/ms]									
APD90 [ms]									
CT KCNJ4									
CT HCN4	0.26439<br>0.3051<br>17								
CT SCN5A	0.31327<br>0.2056<br>18	0.58871<br>0.0012<br>27							
CT CACNA1C	0.39897<br>0.1010<br>18	0.69500<br><.0001<br>27	0.65481<br>0.0001<br>29						
CT KCNH2	0.29053<br>0.2579<br>17	0.58006<br>0.0019<br>26	0.70330<br><.0001<br>28	0.64720<br>0.0002<br>28					
CT CACNA1D	-0.72842<br>0.1006<br>6	0.10944<br>0.8153<br>7	0.71453<br>0.0712<br>7	0.34557<br>0.4477<br>7	0.38486<br>0.3939<br>7				
CT CACNA1G	-0.22706<br>0.6653<br>6	0.56515<br>0.1861<br>7	0.44023<br>0.3229<br>7	0.31254<br>0.4950<br>7	0.28417<br>0.5368<br>7	0.57174<br>0.4283<br>4			
CT KCNA5	-0.70958<br>0.0487<br>8	-0.00728<br>0.9830<br>11	-0.39804<br>0.1780<br>13	0.19096<br>0.5320<br>13	0.06333<br>0.8372<br>13	-0.97117<br>0.0288<br>4	-0.96253<br>0.0375<br>4		
CT KCNJ2	0.39873<br>0.1012<br>18	0.44569<br>0.0256<br>25	0.69364<br><.0001<br>27	0.48381<br>0.0106<br>27	0.55861<br>0.0030<br>26	0.26194<br>0.5704<br>7	-0.53098<br>0.2201<br>7	-0.32365<br>0.2807<br>13	

Supplementary Table S4: Single-cell correlations for atrial Pluricytes. For every pairwise correlation, Pearson´s correlation coefficient r, the p-value and *n* is indicated. For expression parameters (CT), only positive cells are included in the analysis. Significant correlations ($p < 0.05$) are highlighted.

Pearson Correlation Coefficients
Prob > \|r\| under H0: Rho=0
Number of Observations

	Capacitance [pF]	CT TNNT2	CT GAPDH	Beat rate [bpm]	Fast inward current amplitude [nA]	Fast inward current density [nA/pF]	CT KCNJ4	CT HCN4	CT SCN5A	CT CACNA1C	CT KCNH2	CT CACNA1D	CT CACNA1G	CT KCNA5	CT KCNJ2
Capacitance [pF]															
CT TNNT2	-0.40012<br>0.0527<br>24														
CT GAPDH	-0.47483<br>0.0190<br>24	0.92612<br><.0001<br>24													
Beat rate [bpm]	0.05421<br>0.7645<br>33	-0.34275<br>0.1011<br>24	-0.18465<br>0.3877<br>24												
Fast inward current amplitude [nA]	0.13761<br>0.4937<br>27	0.11004<br>0.6638<br>18	0.00055<br>0.9983<br>18	-0.14726<br>0.4636<br>27											
Fast inward current density [nA/pF]	0.44261<br>0.0208<br>27	-0.08424<br>0.7396<br>18	-0.23965<br>0.3382<br>18	0.02145<br>0.9154<br>27	0.87199<br><.0001<br>27										
CT KCNJ4	0.04194<br>0.9084<br>10	0.41908<br>0.2280<br>10	0.38299<br>0.2747<br>10	-0.25614<br>0.4750<br>10	0.81202<br>0.0497<br>6	0.73600<br>0.0953<br>6									
CT HCN4	-0.26246<br>0.2153<br>24	0.83107<br><.0001<br>24	0.82874<br><.0001<br>24	-0.34095<br>0.1030<br>24	-0.02507<br>0.9213<br>18	-0.29049<br>0.2423<br>18	0.46375<br>0.1770<br>10								
CT SCN5A	-0.10878<br>0.6129<br>24	0.59705<br>0.0021<br>24	0.48698<br>0.0158<br>24	-0.42486<br>0.0385<br>24	0.58832<br>0.0102<br>18	0.36252<br>0.1393<br>18	0.22506<br>0.5319<br>10	0.63971<br>0.0008<br>24							
CT CACNA1C	0.01258<br>0.9557<br>22	0.74915<br><.0001<br>22	0.62045<br>0.0021<br>22	-0.37129<br>0.0889<br>22	0.44823<br>0.0816<br>16	0.29919<br>0.2603<br>16	0.45614<br>0.1852<br>10	0.74993<br><.0001<br>22	0.74429<br><.0001<br>22						
CT KCNH2	-0.32193<br>0.1250<br>24	0.91934<br><.0001<br>24	0.93871<br><.0001<br>24	-0.26181<br>0.2165<br>24	0.20871<br>0.4059<br>18	-0.05058<br>0.8420<br>18	0.59746<br>0.0682<br>10	0.87736<br><.0001<br>24	0.69341<br>0.0002<br>24	0.78575<br><.0001<br>22					
CT CACNA1D	-0.02504<br>0.9267<br>16	0.24294<br>0.3646<br>16	0.29601<br>0.2656<br>16	-0.33018<br>0.2117<br>16	-0.35265<br>0.2609<br>12	-0.55552<br>0.0608<br>12	-0.26572<br>0.4895<br>9	0.54085<br>0.0305<br>16	0.22235<br>0.4078<br>16	0.21266<br>0.4291<br>16	0.33062<br>0.2110<br>16				
CT CACNA1G	-0.27952<br>0.3130<br>15	0.36712<br>0.1783<br>15	0.33911<br>0.2163<br>15	-0.50443<br>0.0552<br>15	0.08375<br>0.7958<br>12	-0.12437<br>0.7002<br>12	0.03913<br>0.9267<br>8	0.27949<br>0.3130<br>15	0.58176<br>0.0229<br>15	0.50953<br>0.0524<br>15	0.32717<br>0.2339<br>15	0.02963<br>0.9235<br>13			
CT KCNA5	-0.42523<br>0.0431<br>23	0.87963<br><.0001<br>23	0.74687<br><.0001<br>23	-0.28490<br>0.1876<br>23	0.05977<br>0.8137<br>18	-0.10873<br>0.6676<br>18	0.03325<br>0.9273<br>10	0.72943<br><.0001<br>23	0.58483<br>0.0034<br>23	0.70372<br>0.0004<br>21	0.73649<br><.0001<br>23	-0.13819<br>0.6098<br>16	0.48246<br>0.0685<br>15		
CT KCNJ2	-0.21663<br>0.4570<br>14	0.29410<br>0.3074<br>14	0.16978<br>0.5617<br>14	-0.53080<br>0.0508<br>14	0.35243<br>0.2878<br>11	0.09561<br>0.7798<br>11	-0.65207<br>0.1125<br>7	-<br>0.07961<br>0.7868<br>14	0.48736<br>0.0771<br>14	0.20556<br>0.5005<br>13	0.24389<br>0.4007<br>14	0.63635<br>0.0353<br>11	0.35503<br>0.2840<br>11	0.04165<br>0.8876<br>14	

Supplementary Table S5: Single-cell correlations for primary human ventricular cardiomyocytes**.** For every pairwise correlation, Pearson´s correlation coefficient r, the p-value and *n* is indicated. For expression parameters (CT), only positive cells are included in the analysis. Significant correlations ($p < 0.05$) are highlighted.

Pearson Correlation Coefficients<br>Prob > |r| under H0: Rho=0<br>Number of Observations															
	Capacitance [pF]	CT TNNT2	CT GAPDH	Beat rate [bpm]	Fast inward current amplitude [nA]	Fast inward current density [nA/pF]	CT KCNJ4	CT HCN4	CT SCN5A	CT CACNA1C	CT KCNH2	CT CACNA1D	CT CACNA1G	CT KCNA5	CT KCNJ2
Capacitance [pF]															
CT TNNT2															
CT GAPDH		0.92943<br><.0001<br>66													
Beat rate [bpm]															
Fast inward current amplitude [nA]															
Fast inward current density [nA/pF]															
CT KCNJ4		0.68812<br><.0001<br>60	0.53555<br><.0001<br>60												
CT HCN4		0.83212<br><.0001<br>62	0.79769<br><.0001<br>62				0.69715<br><.0001<br>56								
CT SCN5A		0.93413<br><.0001<br>66	0.86351<br><.0001<br>66				0.70826<br><.0001<br>60	0.83395<br><.0001<br>62							
CT CACNA1C		0.85971<br><.0001<br>66	0.83918<br><.0001<br>66				0.50424<br><.0001<br>60	0.78715<br><.0001<br>62	0.89932<br><.0001<br>66						
CT KCNH2		0.92163<br><.0001<br>66	0.86418<br><.0001<br>66				0.66709<br><.0001<br>60	0.79228<br><.0001<br>62	0.93961<br><.0001<br>66	0.82845<br><.0001<br>66					
CT CACNA1D		-0.50750<br>0.1631<br>9	-0.44537<br>0.2296<br>9				-0.55434<br>0.1214<br>9	-<br>0.90516<br>0.0051<br>7	-0.59789<br>0.0890<br>9	-0.68836<br>0.0404<br>9	-0.56775<br>0.1108<br>9				
CT CACNA1G		-0.38033<br>0.4570<br>6	-0.34388<br>0.5045<br>6				-0.19650<br>0.7090<br>6	-<br>0.51595<br>0.4841<br>4	-0.29833<br>0.5658<br>6	-0.36568<br>0.4759<br>6	-0.28523<br>0.5838<br>6	-1.00000<br>.<br>2			
CT KCNA5		0.66843<br><.0001<br>58	0.55315<br><.0001<br>58				0.49670<br>0.0001<br>55	0.53913<br><.0001<br>54	0.69113<br><.0001<br>58	0.59693<br><.0001<br>58	0.72190<br><.0001<br>58	-0.64581<br>0.0603<br>9	-0.40892<br>0.4208<br>6		
CT KCNJ2		0.91031<br><.0001<br>64	0.89763<br><.0001<br>64				0.54435<br><.0001<br>59	0.77569<br><.0001<br>60	0.91569<br><.0001<br>64	0.88096<br><.0001<br>64	0.86678<br><.0001<br>64	-0.62797<br>0.0702<br>9	-0.16629<br>0.7529<br>6	0.53863<br><.0001<br>57	

Supplementary Table S6: Single-cell correlations for primary human atrial cardiomyocytes. For every pairwise correlation, Pearson´s correlation coefficient r, the p-value and *n* is indicated. For expression parameters (CT), only positive cells are included in the analysis. Significant correlations ($p < 0.05$) are highlighted.

Pearson Correlation Coefficients
Prob > |r| under H0: Rho=0
Number of Observations

	Capacitance [pF]	CT TNNT2	CT GAPDH	Beat rate [bpm]	Fast inward current amplitude [nA]	Fast inward current density [nA/pF]	CT KCNJ4	CT HCN4	CT SCN5A	CT CACNA1C	CT KCNH2	CT CACNA1D	CT CACNA1G	CT KCNA5	CT KCNJ2
Capacitance [pF]															
CT TNNT2															
CT GAPDH		0.64314<br><.0001<br>40													
Beat rate [bpm]															
Fast inward current amplitude [nA]															
Fast inward current density [nA/pF]															
CT KCNJ4		0.71803<br><.0001<br>38	0.52165<br>0.0008<br>38												
CT HCN4		0.65271<br><.0001<br>38	0.70499<br><.0001<br>38				0.43418<br>0.0081<br>36								
CT SCN5A		0.92851<br><.0001<br>40	0.65521<br><.0001<br>40				0.77906<br><.0001<br>38	0.63107<br><.0001<br>38							
CT CACNA1C		0.75201<br><.0001<br>39	0.79858<br><.0001<br>39				0.74720<br><.0001<br>37	0.62625<br><.0001<br>37	0.82354<br><.0001<br>39						
CT KCNH2		0.86837<br><.0001<br>40	0.66604<br><.0001<br>40				0.72826<br><.0001<br>38	0.59691<br><.0001<br>38	0.87227<br><.0001<br>40	0.79846<br><.0001<br>39					
CT CACNA1D		0.40383<br>0.0408<br>26	0.28054<br>0.1651<br>26				0.24616<br>0.2356<br>25	0.22168<br>0.2869<br>25	0.28739<br>0.1546<br>26	0.36581<br>0.0721<br>25	0.28597<br>0.1567<br>26				
CT CACNA1G		0.52913<br>0.0136<br>21	0.52760<br>0.0140<br>21				0.49896<br>0.0251<br>20	0.56495<br>0.0094<br>20	0.56293<br>0.0079<br>21	0.61373<br>0.0040<br>20	0.59517<br>0.0044<br>21	0.50094<br>0.0289<br>19			
CT KCNA5		0.79660<br><.0001<br>40	0.71991<br><.0001<br>40				0.67437<br><.0001<br>38	0.60288<br><.0001<br>38	0.74573<br><.0001<br>40	0.80803<br><.0001<br>39	0.68701<br><.0001<br>40	0.44694<br>0.0221<br>26	0.58801<br>0.0051<br>21		
CT KCNJ2		0.52735<br>0.0019<br>32	0.65323<br><.0001<br>32				0.37481<br>0.0345<br>32	0.38563<br>0.0322<br>31	0.57232<br>0.0006<br>32	0.61276<br>0.0002<br>31	0.56497<br>0.0008<br>32	0.30253<br>0.1712<br>22	0.78242<br>0.0001<br>18	0.58690<br>0.0004<br>32	

Supplementary Table S7: TaqMan gene expression assays used for experiments. Probes span introns whenever possible.

Gene (human)	# TaqMan assay
KCNJ2	Hs00265315_m1
KCNJ4	Hs00544821_m1
HCN4	Hs00175760_m1
SCN5A	Hs00165693_m1
CACNA1C	Hs00167681_m1
CACNA1D	Hs00167753_m1
CACNA1G	Hs00367969_m1
KCNA5	Hs00266898_s1
KCNH2	Hs00165120_m1
TNNT2	Hs00165960_m1
GAPDH	Hs00266705_g1
